# Mathematical Modelling of DNA Replication Reveals a Trade-off between Coherence of Origin Activation and Robustness against Rereplication

**DOI:** 10.1371/journal.pcbi.1000783

**Published:** 2010-05-13

**Authors:** Anneke Brümmer, Carlos Salazar, Vittoria Zinzalla, Lilia Alberghina, Thomas Höfer

**Affiliations:** 1 Research Group Modelling of Biological Systems (B086), German Cancer Research Center, Heidelberg, Germany; 2 Department of Biotechnology and Biosciences, Università degli Studi di Milano-Bicocca, Milan, Italy; 3 BioQuant Center, Heidelberg, Germany; 4 Biozentrum, University of Basel, Basel, Switzerland; University of Texas at Austin, United States of America

## Abstract

Eukaryotic genomes are duplicated from multiple replication origins exactly once per cell cycle. In *Saccharomyces cerevisiae*, a complex molecular network has been identified that governs the assembly of the replication machinery. Here we develop a mathematical model that links the dynamics of this network to its performance in terms of rate and coherence of origin activation events, number of activated origins, the resulting distribution of replicon sizes and robustness against DNA rereplication. To parameterize the model, we use measured protein expression data and systematically generate kinetic parameter sets by optimizing the coherence of origin firing. While randomly parameterized networks yield unrealistically slow kinetics of replication initiation, networks with optimized parameters account for the experimentally observed distribution of origin firing times. Efficient inhibition of DNA rereplication emerges as a constraint that limits the rate at which replication can be initiated. In addition to the separation between origin licensing and firing, a time delay between the activation of S phase cyclin-dependent kinase (S-Cdk) and the initiation of DNA replication is required for preventing rereplication. Our analysis suggests that distributive multisite phosphorylation of the S-Cdk targets Sld2 and Sld3 can generate both a robust time delay and contribute to switch-like, coherent activation of replication origins. The proposed catalytic function of the complex formed by Dpb11, Sld3 and Sld2 strongly enhances coherence and robustness of origin firing. The model rationalizes how experimentally observed inefficient replication from fewer origins is caused by premature activation of S-Cdk, while premature activity of the S-Cdk targets Sld2 and Sld3 results in DNA rereplication. Thus the model demonstrates how kinetic deregulation of the molecular network governing DNA replication may result in genomic instability.

## Author Summary

For a cell to divide into two daughter cells, its genetic information must be accurately duplicated. The large genomes in eukaryotic cells are copied from hundreds or thousands of replication origins to achieve the duplication of the entire DNA in a limited time span. Errors that result in incomplete or multiple copying of parts of the genome can cause cancer in humans. To avoid such errors, the replication origins must be activated coherently across the genome, and repeated firing of already activated origins must be strictly prevented. We developed a kinetic model of the biochemical network that governs the initiation of DNA replication in yeast to understand how these functional properties are realized through the interaction of multiple molecular players. Our computational analysis shows that optimized kinetic parameters are required for the biological functionality of the network, and such parameters indeed account for the measured kinetics of replication initiation. We predict that both the near-synchronous start of replication and the robustness against DNA rereplication are supported by time delays caused by multiple regulatory protein phosphorylations. Our analysis suggests that the kinetic design of the DNA replication network represents an adaptation to multiple, and partially conflicting, functional requirements.

## Introduction

A complex molecular machinery ensures that the entire nuclear genome of eukaryotic cells is replicated exactly once during each cell cycle [1]–[3]. To achieve this, DNA synthesis starts near-simultaneously from hundreds to thousands of replication origins. The assembly of the replication machinery at these origins is separated into two phases, origin licensing and firing. When replication is initiated in the firing phase by cyclin-dependent kinases (Cdks), licensing is inhibited by the same enzymes so that an origin cannot be relicensed, and rereplication of DNA is prevented. Perturbations of the molecular network that governs replication initiation may result in genomic instability through multiple or incomplete replication of parts of the genome [4], [5]. Indeed, many genes implicated in human cancers affect the G1/S transition of the cell cycle [6]. How molecular defects result in aberrant DNA replication is presently not understood in quantitative terms. For example, a model for genomic instability is the *S. cerevisiae* mutant with deletion of Sic1, a cell-cycle regulator that inhibits S-phase Cdk (S-Cdk, formed by kinase Cdc28 and cyclins Clb5,6) [5], [7], [8]. This defect drastically reduces the number of active replication origins [8] but also predisposes cells for DNA rereplication [9]. It is not known how the absence of an endogenous Cdk inhibitor can cause these apparently opposite effects on the activation of replication origins.

Replication dynamics have been studied in detail in yeast [10]–[14]. The activation of several hundred replication origins is not completely synchronous but occurs throughout most of S phase, and not all potential origins fire in each round of replication. However, groups of neighbouring origins and origins in similar regions of the chromosomes (e.g., the centromeric origins in *S. cerevisiae*) fire coherently, and one typically finds a group of ∼200 ‘early’ origins in the yeast genome that are activated rapidly within a ∼10 min interval [10], [11], [15]. Similarly, in early embryonic cell cycles in metazoans thousands of replication origins are activated in a short time interval (10–20 min), while replication initiation in differentiated cells is usually much less coherent [16]. Random fluctuations in assembly rates of replication complexes and effects of the chromatin environment have been invoked to explain the temporally distributed and probabilistic activation of replication origins [17], [18]. Recent computational studies have explored how firing time distributions affect the replication of yeast and *Xenopus* genomes [16], [19], [20]. However, a quantitative understanding of the molecular network that activates the replication origins is lacking, as compared to quantitative analyses of various other aspects of cell-cycle dynamics [21]–[23].

In this paper, we make use of the large body of experimental studies in *S. cerevisiae* to develop a mathematical model of the core regulatory network that governs the initiation of DNA replication. The model implements the molecular mechanisms of origin licensing and its inhibition [1], [3], [24] as well as the recently identified Cdk-dependent mechanisms of origin firing [25]–[27]. We characterize the molecular interactions in the network in terms of measurable kinetic parameters to address the following questions: (1) How rapidly and synchronously can origins be fired, and what are the resulting replicon sizes? (2) How can rereplication be inhibited robustly by the multiple Cdk-dependent mechanisms? (3) Can various experimental findings of genomic instability be rationalized as perturbations of the regulatory network that initiates DNA replication? We find that basic functional requirements on the network dynamics considerably restrict the admissible ranges for the kinetic parameters. Consequently, we choose network parameterizations in an automated manner by constrained optimization. The computational analyses reveal a trade-off between coherent activation of replication origins and robustness against rereplication of DNA.

## Results

### Mathematical model of DNA replication initiation

**Figure 1:**
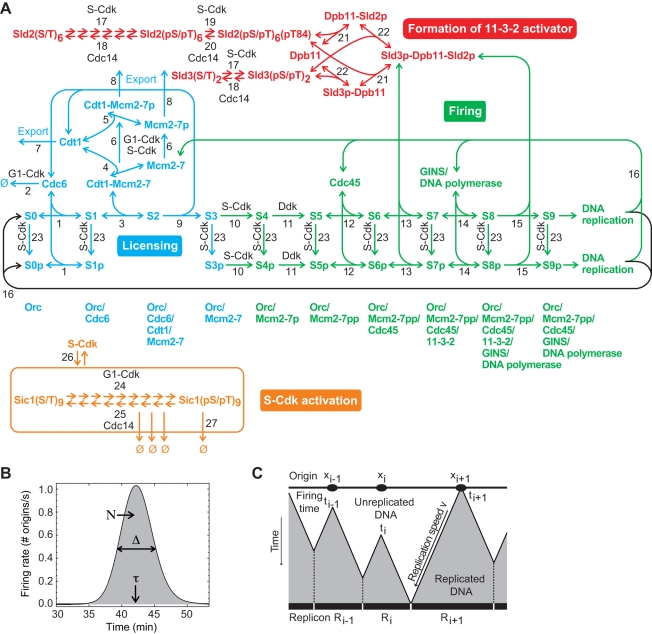
Model of DNA replication initiation. (**A**) The molecular network of DNA replication initiation consists of four modules: licensing of replication origins (blue), activation of S-Cdk (orange), formation of the 11-3-2 activator (red), and origin firing (green), together with multiple mechanisms for inhibiting the relicensing of fired origins, as described in the text. (**B**) The firing rate *f*(*t*), total number of firing origins *N*, mean time to origin firing τ and the duration of origin firing Δ. (**C**) For each origin *i*, the replicon *R_i_* is calculated using the simulated firing times *t_i_*, the origin positions *x_i_* derived from the measured distribution of inter-origin distances and the experimentally determined polymerase progression speed *v* (Text S3).

We have modelled the principal molecular processes that lead to the initiation of DNA replication in *S. cerevisiae*, distinguishing four modules (Figure 1A) [28]. In the licensing phase, pre-replicative complexes (pre-RC) are formed by loading Mcm2-7 proteins (the putative helicases) to the origin-recognition complexes (ORCs), with transient involvement of the licensing factors Cdc6 and Cdt1 [29] (origin licensing module, containing the origin states S0–S3; blue in (Figure 1A). Further progression of replication initiation requires the activation of S-Cdk that is achieved by G1-phase Cdk (G1-Cdk, formed by Cdc28 and cyclins Cln1,2) through phosphorylation of the S-Cdk inhibitor, Sic1, leading to Sic1 degradation [30], [31] (S-Cdk activation module; orange in Figure 1A). G1-Cdk and most S-Cdk (in particular those complexes containing Clb5) remain active until well after completion of S-phase, so that for the time domain of the model only their activation is relevant [1], [32]. Depending on several phosphorylation events, the complete replisome can now assemble (origin firing module, containing origin states S4–S9; green in Figure 1A). Phosphorylation of Mcm proteins by S-Cdk and Ddk promotes the stable binding of Cdc45 (that can more loosely associate with pre-RCs before) [33], [34], and eventually the GINS complex and DNA polymerases bind. The latter two events require the prior activation of the Sld2 and Sld3 proteins that, via the adaptor Dpb11, form the 11-3-2 activator complex [25]–[27]. The 11-3-2 activator is essential for the initiation of DNA replication, but appears to associate only transiently with the replication origins [35]. Mcm proteins, Cdc45, the GINS complex and DNA polymerases are required for DNA synthesis [36], [37]. Sld2 and Sld3 are activated by S-Cdk through multiple phosphorylations [25], [27], [38], [39] (11-3-2 activator module, red in Figure 1A).

In addition to these activating processes, we have modelled multiple mechanisms for inhibiting relicensing as follows: G1-Cdk phosphorylates Cdc6, targeting it for degradation [40]. Free Cdt1 and Mcm2-7 proteins are exported from the nucleus after phosphorylation by G1-Cdk or S-Cdk [41]–[43]. Moreover, S-Cdk inhibits origin (re)licensing by phosphorylating ORC components (Orc2 and Orc6; states labelled with subscript P in Figure 1A) [44], which prevents the Cdt1-dependent loading of Mcm proteins (in state S1P) [45]. Conversely, S-Cdk cannot bind to origins and phosphorylate Orc proteins when Cdt1 is bound [46]. Origins can be licensed as long as Cdc6, Cdt1 and Mcm2-7 are available and licensing has not been inhibited by phosphorylation of Orc proteins.

### Quantitative measures for the dynamics of DNA replication

The state variables *S_i_*(*t*) of the model give the fraction of origins in state *i* at time *t*, which is equal to the probability that an individual replication origin is in state *i*. Accordingly, the *rate of origin activation*, *or firing rate*, (the flux of origins into the replication states S9 and S9P in Figure 1A) is
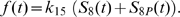
(1)
*f*(*t*) will be the principal output of simulating the dynamics of the replication initiation network (Figure 1B) and can be compared directly to experimental measurements of origin firing in synchronized cell populations [10], [11].

From the moments of the firing time distribution, several characteristics of replication initiation are calculated. The average *number of origin firing events* is
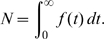
(2)


Rereplication, if occurring, will also contribute to *N*. Therefore we count separately the average *number of rereplicating origins*

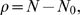
(3)with *N*_0_ being the average number of firing origins, when rereplication is made impossible (*k_16_* = 0 s^−1^). For normal replication, *ρ* must be exceedingly small.

The *mean time to origin firing* (*τ*) is given by
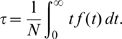
(4)We choose *t* = 0 as the exit from mitosis so that *τ* is the average time from the beginning of licensing to the initiation of replication. The *duration of origin firing* (Δ) is quantified by twice the standard deviation of the firing rate:
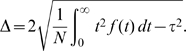
(5)This term gives the mean time interval between the firing of two randomly chosen origins and thus measures the temporal *coherence* of the origin activation events. High coherence of origin firing results in a small value of Δ.

Using the calculated firing rate *f*(*t*), the experimentally measured inter-origin distances, and the synthesis speed of DNA polymerases [10], [15], we compute the total length of the DNA replicons synthesized in 3′ and 5′ direction from each activated origin, with boundaries defined by the points where two counter-propagating replication forks meet (Text S3). The firing times are assigned randomly to the individual origins so that the relevant measure is the *distribution of replicon sizes* (Figure 1C). A narrow size distribution indicates efficient usage of the replication origins.

### Optimized kinetic parameters required for rapid and coherent origin activation

We focus on the set of early-firing origins in *S. cerevisiae* to abstract from not well-characterized processes that may be relevant for late-firing origins (such as less accessible chromatin). As early origins appear to fire largely stochastically, we do not explicitly account for their genomic identity but, to be specific, consider a number of 190 early origins with the measured distribution of inter-origin distances given by Lengronne et al. [15]. However, the computational results in this study are practically independent of the total number of origins within reasonable bounds (Figure S5).

We have translated the reaction scheme of Figure 1A into a set of balance equations for the protein complexes at the replication origins (S and S_P_ states) and the concentrations of unbound (phosphorylated and unphosphorylated) proteins (Text S1). The abundance of the proteins ranges from >100 (Sld3) to several thousand molecules (e.g., Mcm proteins) averaged in an asynchronous cell population [47], and accordingly we have found in sample stochastic simulations that the fluctuations in free protein numbers were negligible compared with the intrinsic random variations in the assembly rates of the protein complexes at the origins. This allowed us to treat the free protein concentrations as deterministic variables. Protein binding events are generally reversible [48], with the exception of rather stable binding of Mcm2-7 to DNA mediated by ATPase activities of Cdc6 and ORC proteins as well as elongating DNA polymerase [24], [29].

We asked for which kinetic parameter the network is functional, showing coherent and rapid activation of replication origins without rereplication. To focus on origin activation, we randomly drew the kinetic parameters for the 11-3-2 activator and firing modules from biochemically feasible ranges [49]. The parameters of the licensing and S-Cdk activation modules were fixed such that licensing of early origins was completed before S-Cdk became active [1] (Table S2). Protein concentrations were taken from Ghaemmaghami et al. [47] (Table S1). Only ∼8% of ∼1000 randomly generated parameter sets realized activation of an appreciable fraction of origins (>95%) without significant rereplication (ρ<0.01, corresponding to less then one rereplication event in 100 cell cycles). Without exception, the random parameters sets showed unrealistically slow kinetics (mean time to firing τ = 3.6±2.1 h and duration of firing Δ = 3.9±2.9 h). This result remained true even when we substantially relaxed the requirement for the fraction of firing origins (accepting parameter sets with only >50% activated origins).

To obtain kinetic parameters that yield a realistic time course of origin firing, we subjected the parameters to a minimization of the duration of firing:
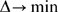
(6)with the constraints that a certain number of origins must fire and do so practically without rereplication:
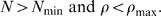
(7)To include variability in the choice of parameters, we did not attempt to find the global optimum of the firing rate but admitted several optimized parameter sets obtained from different initial conditions (Text S2).

**Figure 2:**
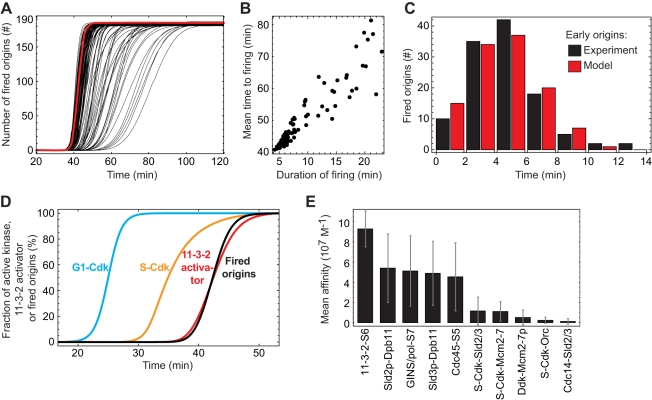
Optimized parameter sets required for rapid and coherent origin activation. (**A**) Admissible parameter sets (*n* = 109) show synchronous activation of replication origins without rereplication (red curve, reference parameter set as given in Table S2). (**B**) Correlation of the duration of origin firing Δ and the mean time to firing τ. (**C**) The distribution of firing times for the early replication origins predicted by the model (red bars, reference parameter set) closely matches the measured replication profile of potential early replication origins in budding yeast (black bars, redrawn from Fig 5, +HU, Yabuki et al. [11]). The simulated distribution was scaled to the same total number of fired origins as in the experiment. (**D**) Typical kinetics of the model (reference parameter set): activation time course of G1-Cdk as an input function (blue curve), followed by delayed activation of S-Cdk (orange curve). S-Cdk triggers formation of the 11-3-2 activator (red curve) and the firing of replication origins (black curve). (**E**) Mean affinities for binding reactions (both binding partners indicated) in the admissible parameter sets. The rather high binding affinities of Cdc45, the 11-3-2 activator and the GINS/DNA polymerase complex to the origins as well as those of phospho-Sld2 and phospho-Sld3 to Dpb11 support fast origin firing. There is hierarchy in regulatory phosphorylations by S-Cdk, with rather high S-Cdk affinity for Sld2 (mean *K*_d_ = 250 nM), and lower for Orc6 (mean *K*_d_ = 1.18 µM). The affinity of the phosphatase for Sld2 and Sld3, counteracting origin firing, is also moderate (mean *K*_d_ = 3.36 µM).

In Figure 2A, we depict the result obtained for 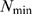 = 180 and 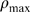 = 0.01. The kinetics of 109 optimized parameter sets agree broadly with the reported kinetics of replication initiation [10], [11], with an average time to firing τ = 49±9 min and firing duration Δ = 9±5 min. The correlation of τ and Δ indicates that there are somewhat ‘faster’ and ‘slower’ parameter sets (Figure 2B). In particular, the faster parameter sets (e.g., red curve in Figure 2A) agree with the observed narrow distribution of replication times for early-firing origins in budding yeast (Figure 2C). The experimentally observed replication kinetics reflects not only those cells that actually fired the origins but also those in which the origin was replicated passively by an incoming fork. However, the model predicts that when the firing duration Δ is small (10 min or less) so that neighbouring origins fire at similar times, the probability of passive replication would be very low (Figure S7). Thus, with optimized kinetic parameters the observed replication times can be taken as a surrogate for the origin firing times and the model reproduces the observed kinetics of replication initiation. Initially Cdk activity is low and replication origins can be licensed. When G1-Cdk activity rises sharply [50], origin licensing is beginning to be inhibited and S-Cdk is activated with a characteristic delay. The firing of the licensed origins is triggered with a further delay provided by the S-Cdk-dependent 11-3-2 activator (Figure 2D). Most parameters in the admissible sets attain preferred values (Figures 2E, S1, S2, and Table S3).

As exact values for the constraints 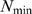 and 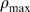 are not known, we repeated the optimization of firing rate with different values. We found that the permitted (small) probability of rereplication 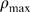 can serve as a stringent constraint for fast origin firing (especially for very small 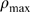). By contrast, the required number of firing origins 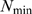 does not strongly constrain the optimization of firing. Even when we required only 50% of firing origins (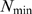 = 95), we found that on average ∼90% of origins (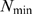 = 170) fired in the optimized parameter sets (Figure S6).

In summary, optimized kinetic parameters of the origin firing and 11-3-2 activator modules give rise to realistic kinetics of origin firing.

### Network robustness: rapid origin firing carries increased risk of rereplication

**Figure 3:**
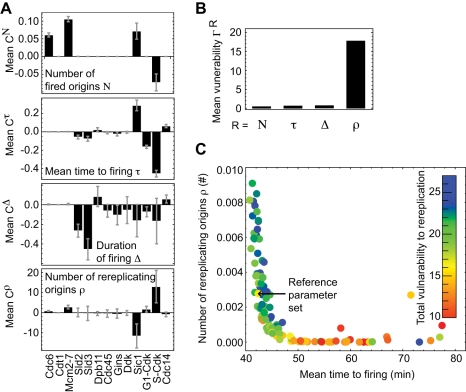
Sensitivity to variations in protein concentrations. (**A**) Control coefficients for the total number of fired origins *N*, the mean time to firing t, the duration of firing Δ, and the number of rereplicating origins ρ, calculated for the concentration of each protein component (mean value and standard deviation for admissible parameter sets). (**B**) Total number of firing origins *N*, mean time to firing τ and the duration of firing Δ are robust network responses, whereas the number of rereplicating origins ρ is very vulnerable against fluctuations in the protein concentrations. (**C**) Fast origin activation increases the probability for rereplication. Colour code indicates the vulnerability to rereplication.

The expression of the proteins in the network may vary from cell to cell. Moreover, the protein numbers were measured in asynchronous populations and may be different at the G1/S transition [47]. To quantify the effect of variations in protein abundance on the systems performance, we calculated the control coefficients for the systems responses *R* with respect to the protein concentration *X_i_*

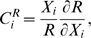
(8)where *R* = *N*, τ, Δ and ρ. Rereplication (ρ) is at least one order of magnitude more sensitive to concentration variations than the other responses and has unusually high control coefficients (Figure 3A; e.g., 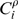 = 10 indicates a 100% change in the number of rereplicating origins if concentration of protein *i* is varied by 10%). A decrease in the concentration of Sic1 and an increase in S-Cdk concentration cause the largest rise in the probability for re-initiating replication from a fired origin. Practically all proteins in network have some control on the number of activated origins and firing kinetics, but compared with rereplication, the control coefficients are an order of magnitude smaller (Figure 3A). Analogous results were obtained for the control by kinetic parameters (Figure S3).

The high vulnerability of the network to rereplication is clearly seen by quantifying the overall sensitivity of a systems response by computing the norm of the control-coefficient vectors:
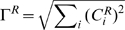
(9)(Figure 3). This result implies that the absolute probability of rereplication must be kept exceedingly small to tolerate variations in protein numbers without rereplication actually occurring.

We further asked whether there are systematic relations between the different network responses. We observed a strong nonlinear correlation between the risk of rereplication and the kinetics of origin firing, with the (very small) number of rereplicating origins increasing as firing becomes faster and more synchronous (in Figure 3C shown for *ρ* vs *τ*; the graph *ρ* vs Δ looks very similar). Moreover, there exist parameter sets for which origin firing is even faster but for all of these rereplication exceeds the assumed cut-off *ρ* = 0.01 (not shown). Moreover, networks that fire fast and coherently are also somewhat less robust to rereplication (colour code for overall sensitivity 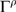 in Figure 3C). This higher vulnerability is primarily due to the increased control coefficients of S-Cdk and Sic1 (Figure 3A, 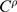). Thus the requirement for robustness of the network against DNA rereplication imposes a limit on the speed and coherence of origin firing.

### Premature activation of S-Cdk or Sld2 and Sld3 strongly perturbs replication initiation

**Figure 4:**
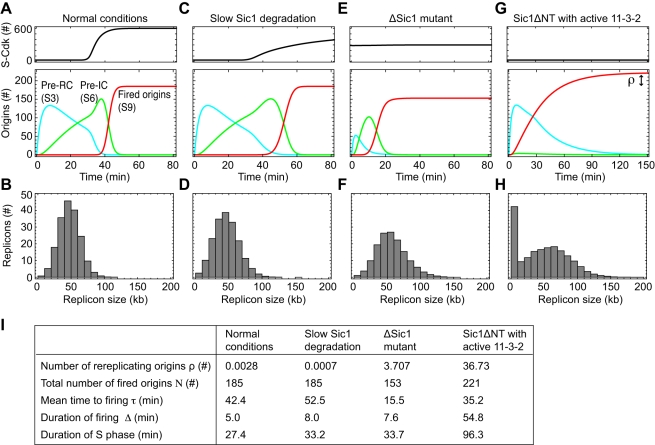
Deregulation of S-Cdk activity reduces origin firing and causes rereplication. (**A**) Rapid activation of S-Cdk (upper panel) causes coherent origin firing (lower panel, red curve). Blue and green curves show the kinetics of pre-replicative and pre-initiation complexes. (**B**) Distribution of replicon size corresponding to the firing kinetics in (A). (**C**) Slow activation of S-Cdk (upper panel) due to a slow Sic1 degradation, as observed under suboptimal growth conditions, causes delayed origin firing (lower panel, red curve). (**D**) Distribution of replicon size corresponding to the firing kinetics in (C). (**E**) Premature and reduced activation of S-Cdk, as suggested for the *Δsic1* mutant (upper panel) causes reduced origin licensing and premature origin firing (lower panel). (**F**) The distribution of replicon sizes corresponding to firing kinetics of (E) is broadened and large replicons (>120 kb) can occur. (**G**) A constitutive 11-3-2 activator together can bypass the requirement for S-Cdk (using a non-degradable mutant form of Sic1, *sic1*ΔNT) in replication initiation but causes asynchronous firing and considerable rereplication. (**H**) Distribution of replicon size corresponding to the firing kinetics in (G). (**I**) Quantification of the kinetics of origin firing for the conditions shown in (A)–(H). All simulations with reference parameter set; parameter changes for (C)–(H) specified in Text S2.

Sic1 and S-Cdk emerged as the components with major control on DNA rereplication (followed by Mcm2-7; cf. Figure 3A). Under typical growth conditions, S-Cdk is rapidly activated following the degradation of Sic1 (Figure 4A, upper panel) and replication is triggered at nearly all simulated origins (185 of 190) in a coherent manner (Δ = 5 min) (Figure 4A, lower panel; cf. Figure 2D). The computed distribution of replicon sizes centers narrowly about the average spacing of 46 kb (Figure 4B). In particular, short (<5 kb) and very long (>120 kb) replicons do not occur, implying that the triggered replication origins all contribute appreciably to DNA synthesis. Suboptimal growth conditions prolong the S phase in budding yeast [51], [52], possibly because of slower degradation of Sic1 [53]. In the model, this leads to slower accumulation of active S-Cdk (Figure 4C, upper panel). The onset of origin firing is delayed and more desynchronized (Δ = 8 min) (Figure 4C, lower panel), and the distribution of replicon sizes slightly broadens (Figure 4D). There is a small probability of passive replication of the later-firing origins. Importantly, however, the probability of rereplication is extremely small irrespective of whether S-Cdk is activated rapidly or in a delayed manner (Figure 4I). Thus the model is robust with respect to the activation kinetics of S-Cdk.

In the *Δsic1* mutant, S-Cdk is activated prematurely and to a lesser extent [8], [52]. Simulating this situation, we find that licensing cannot be completed at a sizable fraction of replication origins because S-Cdk-dependent mechanisms that inhibit licensing set in too early (Figure 4E; 22% of unlicensed origins in the model, compared with 25% estimated from experimental data by Lengronne & Schwob [8]). This gives rise to a broadened distribution of replicon sizes with increased distances between firing origins (Figure 4F), as observed experimentally [8]. Some licensed origins are activated very rapidly because S-Cdk is already active before pre-initiation complexes (pre-ICs) are formed (*τ* = 15 min) and a small number of origins (ρ = 3.7) is activated so early that they fire twice. Such a small degree of rereplication (2%) is at the detection limit of currently available experimental methods [54]. Moreover, rereplication does not occur in several other admissible parameter sets (not shown). However, our results indicate that the *Δsic1* mutant is more prone to rereplication than the wildtype. This is in agreement with findings by Ikui et al. [9] that the lack of Sic1 in the presence of further mutations (which prevent inhibitory Orc phosphorylations by S-Cdk and degradation of Cdc6) is lethal, probably due to premature origin firing and rereplication.

To analyse the effect of premature origin firing, we simulated experiments with constitutively active Sld2 and Sld3, so that DNA replication is initiated without the need for S-Cdk-dependent phosphorylation events (Figure 4G). In the case of strongly reduced S-Cdk activity, origin firing starts much earlier than in the ‘wildtype’ model (and also earlier than in the *Δsic1* mutant) and is strongly desynchronized (Δ = 55 min, including rereplication), Moreover, in agreement with the experimental data there is massive rereplication (∼19% of origins) [25], [27]. As a consequence, the distribution of replicon sizes is very broad, with replicon sizes up to 200 kb and many (∼17%) passively replicated origins (Figure 4H). Rereplication appears to be due to both premature origin firing and the lack of licensing inhibition by S-Cdk.

**Figure 5:**
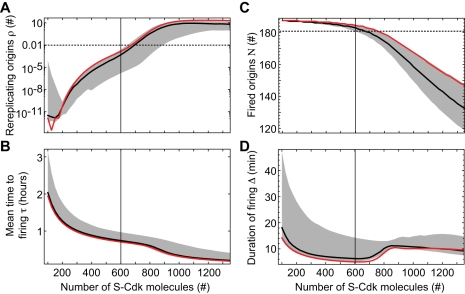
Optimal S-Cdk activity for efficient origin firing without rereplication. (A) Number of rereplicating origins ρ, (B) mean time to firing τ, (C) number of fired origins *N*, and (D) the duration of firing Δ, each versus number of active S-Cdk molecules. Black curve, median of all admissible parameter sets; gray area, 68% quantile; red curve, reference parameter set.

To study the effect of S-Cdk abundance, we varied the number of S-Cdk molecules (with unchanged Sic1 regulation by G1-Cdk) and observed that over a broad range of active S-Cdk concentration, in the model corresponding to ∼200–800 molecules in the nucleus, origin activation is fast and without rereplication (Figure 5). Moreover, the distribution of firing times, and thus, coherence of origin firing, is not considerably affected by a strong reduction in the number of S-Cdk molecules, as occur when Clb5 is deleted (Figure S8) [55]. However, the replication pattern is altered at extreme S-Cdk concentrations. With a very small number of S-Cdk molecules (<100), origin firing will be slower and less coherent. For very high numbers (>900), fewer origins will fire because licensing is inhibited more rapidly by S-Cdk dependent Orc phosphorylations. In parallel, rereplication may occur from a small number (<10) of origins. Moreover, we find that too low S-Cdk activity also causes DNA rereplication at least in some parameter sets, indicating that tight inhibition of origin (re-)licensing strictly requires S-Cdk-dependent phosphorylation events (not shown). This result is in agreement with experimental data showing that transient overexpression of Sic1 in G2-arrested cells causes loss of Orc6 phosphorylation and DNA rereplication once Sic1 levels begin to drop again [5].

In summary, the model indicates that replication is robust for a considerable range of S-Cdk activation kinetics and concentration. However, premature activation of S-Cdk causes a strong reduction in the number of firing origins, while premature activity of the S-Cdk targets Sld2 and Sld3 can result in DNA rereplication.

### Multisite protein phosphorylation supports coherent origin activation without rereplication

**Figure 6:**
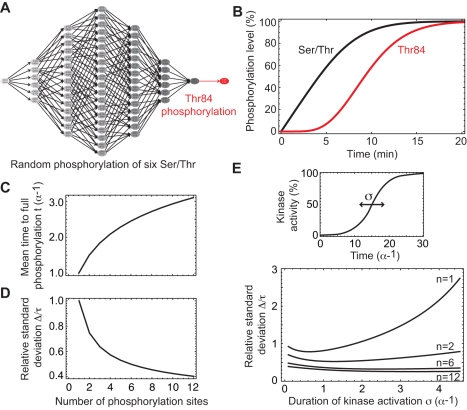
Mechanism of multisite protein phosphorylation. (**A**) Mixed random-sequential phosphorylation of Sld2: six Ser/Thr residues are phosphorylated randomly, giving rise to a large number of possible phosphorylation pathways (states 0: unphosphorylated and 1: phosphorylated). Together, these phosphorylations cause a conformational change that allows phosphorylation of the essential Thr84 (state indicated in red), the docking site for Dpb11. (**B**) In agreement with experimental data [38], the model gives rise to hyperbolic time course for each of the initial six Ser/Thr residues (black curve), while Thr84 (red curve) is phosphorylated with a time delay. To mirror the *in-vitro* assay conditions, the model simulation was performed without phosphatase activity. (**C**) The mean time for complete phosphorylation rises with the number of phosphorylation sites (the increase is less than linear because of the random phosphorylation mechanism), while (**D**) the temporal coherence of phosphorylation increases (decreasing Δ/*τ*). (**E**) The robustly high temporal coherence of protein activation by multisite phosphorylation (low Δ/*τ*) contrasts with the strong dependence of single-site phosphorylation on the activation time of the input kinase, σ (see upper panel).

Next we studied in more detail how the activation of Sld2 and Sld3 is timed. Multisite protein phosphorylation is a conspicuous element of both S-Cdk activation and 11-3-2 activator formation. The rapid degradation of Sic1 appears to require six or more phosphorylations by G1-Cdk, and activation of Sld2 and Sld3 by S-Cdk involves seven and two phosphorylation steps, respectively [27], [31], [38], [39]. To focus on a specific process, we have modelled the reversible phosphorylation of Sld2 by S-Cdk according to the data by Tak et al. [39]. Six serine and threonine residues are assumed to be phosphorylated randomly by S-Cdk; through a conformational change of the protein, this exposes Thr84 for phosphorylation (Figure 6A). Dpb11 eventually binds to phosphorylated Thr84, which mediates the trigger function of Sld2 for replication initiation. Consistent with the comparatively slow kinetics of Sld2 phosphorylation, S-Cdk was assumed to act in a distributive manner, binding and dissociating repeatedly before all residues become phosphorylated [56]. In agreement with *in vitro* measurements, the model yields identical time courses for the first six phosphorylations and delayed phosphorylation of Thr84 (Figure 6B; cf. Tak et al. [39]). An alternative model with processive action of S-Cdk cannot reproduce the experimental data (not shown). Multisite phosphorylation of Sld3 by S-Cdk and of Sic1 by G1-Cdk were modelled in a similar manner as a random, distributive processes (Text S1).

The average time needed to fully phosphorylate a protein rises with the number of phosphorylation sites (Figure 6C), while the duration of the transition to full phosphorylation, measured by Δ/τ, decreases and thus the temporal coherence of activation increases (Figure 6D). In addition, we found that a multisite phosphorylation module also decouples the coherence of the output from temporal variations in the input kinase activity. While protein activation by a single phosphorylation responds coherently to fast kinase activation and uncoherently to piecemeal kinase activation (and thus mirrors the kinase activation kinetics), multisite phosphorylation always results in coherent activation (Figure 6E). Thus distributive multisite phosphorylation mediates both delayed and sharp activation of the target protein regardless of how fast kinase activity is switched on.

**Figure 7:**
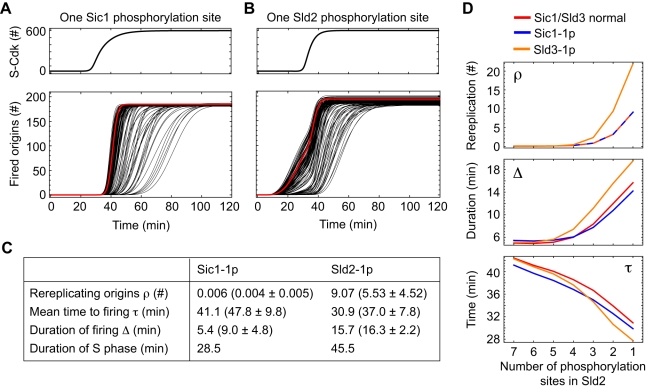
Multisite phosphorylation of Sld2 functions supports coherent origin firing without rereplication. (**A**) Effects of hypothetical single phosphorylation of Sic1 for its fast degradation: less coherent S-Cdk activation (upper panel) but unaffected kinetics of origin firing (lower panel; red curve, reference parameter set; black curves, other admissible parameter sets). (**B**) The effect of reducing the number of phosphorylation sites in Sld2 to a single site is more pronounced. Origin firing starts earlier and the number of rereplicating origins is increased (lower panel) for all parameter sets. (**C**) Number of rereplicating origins ρ, mean time to firing τ, duration of firing Δ, and the length of S phase computed for the reference parameter set (red curves in (A) and (B)). Mean values and standard deviations for all admissible parameter sets (black curves in (A) and (B)) are given in brackets. (**D**) A hypothetical reduction in the number of phosphorylation events required to activate Sld2 strongly affects origin firing (rereplicating origins ρ, firing duration Δ, mean time to firing τ). There is an additional effect of the reduction of required Sld3 phosphorylation steps (from two to one), whereas a reduction in the required Sic1 phosphorylation steps (from six to one) has no additional impact.

These kinetic properties appear suited for replication initiation where robust time delays and coherence are required. To investigate the role of multisite phosphorylation in the replication-initiation network, we varied the number of phosphorylation sites in Sic1, Sld2 and Sld3. The hypothetical assumption that a single phosphorylation of Sic1 is sufficient to trigger its degradation caused less coherent and slightly earlier activation of S-Cdk (Figure 7A, upper panel) but did not alter the network behaviour appreciably for the majority of parameter sets (Figure 7A, lower panel). The values for rereplication, mean firing time, duration of firing were almost unchanged compared to the standard model with six or more Sic1 phosphorylations. By comparison, the effect of a hypothetical reduction in phosphorylations steps for activation of Sld2 to a single step was much more pronounced (Figure 7B). Although S-Cdk activation was unaffected, origins fired prematurely, and a small number (on average 5.5 origins) showed rereplication (Figure 7C).

To analyze this in more detail, we reduced the number of phosphorylations needed to activate Sld2 in a stepwise manner and found a continuous increase in the probability of rereplication that became particularly pronounced for three or fewer phosphorylation steps (Figure 7D). Assuming, in addition, Sic1 degradation after a single phosphorylation did not noticeably increase the probability of rereplication, while Sld3 activation after a single phosphorylation did. Thus multisite phosphorylation of the 11-3-2 activator components Sld2 and Sld3 is critical for a robust suppression of rereplication. As expected from the results shown in Figure 6, reducing the number of phosphorylation events needed to activate Sld2 and Sld3 caused premature entrance into S phase (reduced *τ*) and strongly desynchronized origin firing (increase in Δ) (Figure 7D) and, because of the latter, would also lead to a prolonged S phase (46 min as compared to 27 min under reference conditions; Figure 7C).

Taken together, the time delays imposed by multisite phosphorylations of Sld2 and, to a lesser extent, of Sld3 and Sic1, are critical in the model to time origin firing in such a way that the Cdk-dependent mechanisms of preventing origin relicensing take full effect and rereplication is tightly inhibited. Moreover, multisite phosphorylation of Sld2 supports the coherent firing of replication origins by providing a sharp trigger for initiating DNA replication.

## Discussion

### Optimization of network function

We have developed a mathematical model of the molecular network that initiates DNA replication in *S. cerevisiae* and used an optimization approach for its parameterization. Optimization of objective functions describing systems functionality has previously been employed to rationalize the distribution of kinetic parameters and fluxes in metabolic pathways [57], [58]. The starting point for our approach was the observation that with randomly chosen (biochemically realistic) parameters the replication-initiation network showed unrealistic, too slow and uncoherent kinetics of origin activation. The maximization of firing coherence, constrained by a minimal number of firing origins and non-occurrence of DNA rereplication, yielded network behaviour that accounts for the observed activation kinetics of the early replication origins (roughly half of the activated origins in *S. cerevisiae*). Thus the model captures basic characteristics of rapid replication initiation. The timing of ‘late’ origins is thought to be shaped by additional factors not included in the model, such as decreased chromatin accessibility (see below).

The kinetic design of the network achieves simultaneously coherent origin activation and prevention of DNA rereplication. Control analysis showed that the system is most vulnerable with respect to rereplication, of all functional properties studied. The model accounts for multiple mechanisms inhibiting relicensing of replication origins. Because of the high sensitivity of DNA rereplication to fluctuations in protein concentrations, the various mechanisms inhibiting origin licensing in S and G2 phases, which rely on continued Cdk activity, may not be redundant but might all be needed to confer the desired degree of robustness to the network. Recent experimental work actually indicates that the often presumed independence of the various inhibitory mechanisms needs to be re-evaluated with more sensitive experimental methods [54].

Parameter sets leading to very fast and coherent origin activation are usually associated with a higher probability of, and vulnerability towards, DNA rereplication. This does not seem to constitute a serious problem, as we obtain parameter sets with origin firing as fast as seen in the experimental data, and these have very small probability of rereplication. Nevertheless, the model indicates that a further increase in firing rate and coherence may be impeded by the need to prevent rereplication. Therefore, the dynamics of the network appear to be shaped, at least in part, by conflicting requirements of coherence and robustness against DNA rereplication.

### Timing and probability of origin activation

In budding yeast, replication origins appear to be used with rather high efficiency; on average each origin fires every other cell cycle (50%), and many early origins fire in as many as 90% of S phases [59]–[63]. In fission yeast, the average origin firing efficiency in mitotic cell cycles is lower (∼30%), with early origins also activated more frequently than late ones [12]. Our computational analysis implies that considerable variability in the timing of origin activation is due to rate fluctuations in the assembly of replication complexes and the triggering of DNA synthesis. In particular, the model reproduces the measured activation kinetics of early replication origins in *S. cerevisiae* [10], [11] with optimized kinetic parameter sets (fast activating and slower inhibiting phosphorylations by S-Cdk and several high-affinity protein-protein interactions involved in the activation of replication origins). Interestingly, parameter optimization did not yield biochemically realistic parameter sets that lead to more coherent origin activation than observed experimentally for early origins when the probability of rereplication was required to be very small. In the very fast early embryonic cell cycles in *Xenopus* most replication origins fire within ∼10 minutes [16], which is also in the same order of firing coherence found in the model.

In the model, licensed origins will practically always be biochemically competent to fire. The origin firing efficiency can be modified by passive replication from polymerases coming from neighbouring origins. Factors that reduce firing coherence (e.g., differences in local chromatin state [17]) will increase the probability of passive replication and thus also reduce origin firing efficiency. However, this is unlikely to alone explain firing efficiencies of 30% and less per origin, indicating that important limitations to origin usage occur in the licensing phase and affect origin firing competence. Low affinities of initiation factors or early activation of licensing inhibitory mechanisms due to premature activation of S-Cdk may affect the pre-RC assembly and, thus, reduce the firing competence in the model. A recent study in fission yeast has indeed found that differential recruitment of ORC components and timing of pre-RC assembly correlates with the origin efficiency and the firing time [64].

### Temporal separation between licensing and firing of replication origins

Of the functional constraints the model was subjected to, rereplication was by far most sensitive to parameter changes (although multiple mechanisms of suppressing rereplication were implemented). In particular, we have found that the dual role of Cdk in triggering origin firing and inhibiting licensing *per se* is not enough to prevent the reactivation of fired origins. Rather, there must be a robust time delay before replication is initiated from licensed origins by S-Cdk, because the sufficient depletion of Mcm2-7, Cdt1 and Cdc6 from the nucleus triggered by G1-Cdk and S-Cdk requires time, and S-Cdk activity must become high enough to inhibit origin relicensing by phosphorylation of ORC proteins.

The time delay between the loading of Mcm2-7 to replication origins and origin firing (∼20 min or longer in the model) is controlled primarily through (1) the G1-Cdk-dependent phosphorylation of Sic1, followed by Sic1 degradation, and (2) the phosphorylation of Sld2 and Sld3 by S-Cdk. In support of this result, massive rereplication is observed when the Sld2- and Sld3-dependent time delay is abolished through the introduction of constitutively active Sld2 and Sld3 constructs [25], [27]. Although these experiments were done in a mutant with much reduced S-Cdk activity (because of stabilized Sic1) so that several, but not all, mechanisms of inhibiting rereplication were compromised, we would predict substantial rereplication also in the presence of normal S-Cdk activity. Conversely, the lengthening of the time delay between origin licensing and firing, as observed for example under suboptimal growth conditions [51], [52] or in various mutants [65], [66], has generally no detrimental effect on genomic integrity.

### Distributive multisite phosphorylation of Sld2 by S-Cdk as a robust timing device

Multisite protein phosphorylations of Sic1 by G1-Cdk, Mcm proteins by S-Cdk and Ddk, as well as Sld2 and Sld3 by S-Cdk are a conspicuous feature of replication initiation. The available measurements in *S. cerevisiae* support the temporal order of phosphorylation events observed with optimized parameters in the model, with phosphorylations of Mcm4, Sld3 and binding of Cdc45 occuring before (0–27% of cells budded) phosphorylation of the critical Thr84 in Sld2 (11–57% of cells budded) [25], [34], [39].

Transcriptional positive feedback in G1-Cdk activation has been shown to support switch-like entry into the cell cycle [50]. However, there are sources of variability downstream of G1-Cdk, such as in the regulation of Sic1, that might cause incoherent origin firing. Such variability can be buffered by Sld2 phosphorylation, which also has a strong impact on robustness against rereplication. The distributive and hierarchical phosphorylation of Sld2 causes a time delay between the onset of S-Cdk activation and origin firing that, in turn, allows efficient suppression of re-licensing by S-Cdk. The Sld2 phosphorylation kinetics also provide a sharp trigger for starting DNA replication and decouple coherent origin firing from rate fluctuations in the assembly of the pre-ICs [67]. Thus Sld2 multisite phosphorylation may serve as a ‘decoupling module’ previously hypothesized to support network robustness [68]. Kinetic studies with phospho-site substitution mutants of Sld2 that sensitively monitor rereplication and coherence of origin firing may be used to further examine these theoretical predictions [38], [39]. Counteracting phosphatase(s), such as Cdc14, have little control in the model, which appears to be in agreement with experimental data [69].

While Sic1 is subject to distributive phosphorylation by G1-Cdk [31], the requirement of a large number of phosphorylations for Sic1 degradation and DNA replication has been questioned by recent studies [66], [70]. Interestingly, the time delay associated with Sic1 degradation is of negligible importance in the model for robustness against DNA rereplication, compared to the time delay associated with Sld2 and Sld3 phosphorylations. Mcm proteins are also phosphorylated at multiple sites by S-Cdk and Ddk, mediating stable association of Cdc45 [34], [71]. In contrast to Sld2 and Sic1, Mcm phosphorylations are processive and thus much faster. This may be functionally relevant, as in the model slower Mcm phosphorylations (e.g., through limiting Ddk activity) will result in desynchronized origin firing. Indeed, mutations in Mcm proteins that impair Ddk processivity and delay Mcm phosphorylation cause a very slow progression of the mutant cells through S phase [34].

### Stoichiometric versus ‘catalytic’ factors in replication initiation

Individual proteins may bind in somewhat different order to form pre-ICs, and there appear to be some differences between *S. cerevisiae* and *S. pombe* [2], [3]. We found that such suggested variations in the binding order of proteins (e.g., active Sld3 binding first to Cdc45 while Dpb11 and Sld2 are recruited later to form the complete 11-3-2 activator at the DNA) [35], [72] had practically no effect on the kinetics of origin firing in the model. By contrast, the release of the 11-3-2 activator from replication origins after replication start is critical for coherent origin firing. Stable association of the 11-3-2 activator without release would cause incomplete origin firing (Figure S4) because of the reported rather low number of Sld3 molecules (∼130 [47]). Thus we suggest that the 11-3-2 activator serves a transient ‘catalytic’ function for origin activation in a similar way as Cdc6 and Cdt1 do for origin licensing. While Cdc6 and Cdt1 function relies on their ATPase activity, the underlying molecular mechanism has not been identified for the 11-3-2 activator. Generally, the protein abundance measurements in *S. cerevisiae* [47] support a distinction between stably bound factors (Mcm, Cdc45, GINS) with a few thousand molecules and transient factors (Sld2/3, Dpb11, Clb5/6) with a few hundred copies per cell (although there is the caveat that unsynchronized cell populations were used).

### Genomic instability through deregulation of Sic1

DNA rereplication is a candidate mechanism for genomic instability that may lead to cancer in humans [6]. We have found here that very low S-Cdk activity will lead to delayed and uncoherent onset of DNA replication, while premature S-Cdk activity will cause a reduced number of activated origins and may also predispose to rereplication. In the *S. cerevisiae Δsic1* mutant S-Cdk is prematurely activated in G1 phase. In agreement with the model, a substantial fraction of origins is not licensed because premature S-Cdk activity inhibits licensing [8]. However, it has remained unclear whether the resulting large replicon sizes are ultimately the cause of the incomplete chromosome separation and genomic instability observed in this mutant. The model indicates that this mutant is also less robust with respect to DNA rereplication. This computational result is consistent with the observation that the *Δsic1* strain is more susceptible to rereplication than the wildtype when additional perturbations are applied [9]. Such small probability of rereplication predicted by the model for the *Δsic1* mutant could be experimentally tested by using more accurate methods for detecting rereplication. The model can also account for other scenarios of DNA rereplication, e.g., transient overexpression of Sic1 [5].

Many components of the molecular network that initiates DNA replication, including Sld2, are conserved from yeast to higher eukaryotes [26]. In this paper, we have uncovered kinetic constraints for Cdk activation and origin firing that allow efficient replication initiation to be reconciled with robustness against rereplication. We expect that these design principles hold more generally and help rationalize mechanisms of genomic instability arising through deregulation of the replication-initiation network.

## Methods

### Mathematical model

The mathematical model was formulated as a system of balance equations for the protein complexes assembling at the replication origins and the free protein concentrations, accounting also for different phosphorylation states and protein complexes (Text S1). The initial molecule concentrations were taken from experimental measurements [47] (Table S1). The model equations were solved numerically using Mathematica.

### Determination of kinetic parameter sets

Kinetic parameter values are determined by an optimization approach, in which the functionality of the system is optimized as a function of the kinetic parameters. The optimization algorithm was implemented in Mathematica using the function NMinimize with the option DifferentialEvolution. The detailed procedure of the parameter optimization is explained in the Text S2.

### Calculation of DNA replicon sizes

DNA replicon sizes were calculated by drawing the firing times of individual origins from the computed firing time distribution (Text S3). All computations were performed using Mathematica.

### Note added in proof

After the final version of this paper was submitted, a paper [73] appeared that proposes a mechanism for the RC assembly in which phosphorylated Sld2 forms a “preloading complex” with Dpb11, GINS and DNA polymerase ε before being recruited to the DNA. Sld3 would then bind to Cdc45 prior to these events. We have previously analysed a variant of the model in which phospho-Sld3 associates with Cdc45 before fully phosphorylated Sld2 together with Dpb11 binds and forms the complete 11-3-2 activator complex at the DNA. We have found the main conclusions of the model to be unaffected by such variations in the order of RC assembly.

## Supporting Information

Text S1 Mathematical model
(0.21 MB PDF)

Text S2 Determination of kinetic parameter values
(0.04 MB PDF)

Text S3 Calculation of replicon sizes
(0.05 MB PDF)

Table S1 Protein numbers and concentrations
(0.03 MB PDF)

Table S2 Kinetic parameters of the mathematical model
(0.04 MB PDF)

Table S3 Mean value and standard deviation of optimized parameters
(0.02 MB PDF)

Figure S1 Distribution of optimized parameter values of the firing module
(0.06 MB PDF)

Figure S2 Distribution of optimized parameter values of the 11-3-2 activator module
(0.05 MB PDF)

Figure S3 Control coefficients of the kinetic parameters on the systems properties N, ρ, τ and Δ calculated with all generated parameter sets
(0.05 MB PDF)

Figure S4 Initiation of DNA replication without catalytic replacement of the 11-3-2 activator complex
(0.04 MB PDF)

Figure S5 Dependence of the systems properties on the initial number of early origins calculated with the reference parameter set
(0.03 MB PDF)

Figure S6 Average fraction of activated origins and average number of rereplicating origins in parameter sets generated under different requirements for the constraints N_min_ and ρ_max_.
(0.04 MB PDF)

Figure S7 Estimated number of replicated origins in dependence of the coherence of origin firing
(0.03 MB PDF)

Figure S8 Firing rate for different S-Cdk concentrations
(0.04 MB PDF)
