# Supplementary material for: Mathematical Modelling of DNA Replication Reveals a Trade-off between Coherence of Origin Activation and Robustness against Rereplication
Source: PLoS Comput Biol. 2010 May 13;6(5):e1000783. doi: 10.1371/journal.pcbi.1000783 (PMC2869307; doi:10.1371/journal.pcbi.1000783)
Supplement: Text S1 — Mathematical model (0.21 MB PDF) [file pcbi.1000783.s001.pdf]

Supporting Text 1: Mathematical model

The mathematical model considers the major molecular events leading to the assembly of the replicative complexes at 190 early origins of replication (see Figure 1A of the main text for a scheme of the network). Specifically, we modeled the formation of two replication complexes at each origin promoting the synthesis of the DNA strand in two opposing directions. We assumed that both replisomes are assembled quasi simultaneously (as two consecutively formed replication complexes) and start DNA synthesis synchronously as soon as the second replication complex at an origin is completed. The model consists of a system of ordinary differential equations, describing the time evolution of all the core variables constituting the replication-initiation network. A Mathematica file with the model equations is available on request.
Symbols used for the model variables in the differential equations are shown in the following table.

**Variable**	**Symbol**
States of replication origins	
Orc	S_0
Orc/Cdc6	S_1
Orc/Cdc6/Cdt1/Mcm2-7	S_2
Orc/Mcm2-7	S_3
Orc/Mcm2-7/S-Cdk	S_{Cdk}
Orc/Mcm2-7p	S_4
Orc/Mcm2-7p/Ddk	S_{Ddk}
Orc/Mcm2-7pp	S_5
Orc/Mcm2-7pp/Cdc45	S_6
Orc/Mcm2-7pp/Cdc45/11-3-2	S_7
Orc/Mcm2-7pp/Cdc45/11-3-2/GINS/DNA polymerase	S_8
Orc/Mcm2-7pp/Cdc45/GINS/DNA polymerase	S_9
Orc-S-Cdk/... for i=0,1,3,4,5,6,7,8,9	Sc_i
Orcp/... for i=0,1,3,4,5,6,7,8,9	Sp_i
Binding factors	
Cdc6	F_1
Cdt1	F_{2a}
Mcm2-7	F_{2b}
Mcm2-7p	F_{2bp}
Cdt1-Mcm2-7	F_2
Cdt1-Mcm2-7p	F_{2p}
Cdc45	F_4
GINS/DNA polymerase	F_5
Sic1 degradation, Cdks and phosphatase	
Free Sic1 with i phosphorylations	Sic_i
S-Cdk bound Sic1 with i phosphorylations	Z_i
G1-Cdk bound S-Cdk/Sic1 with i phosphorylations	Za_i
Cdc14 bound S-Cdk/Sic1 with i phosphorylations	Zb_i
Clb5,6-Cdc28 (S-Cdk)	Cdk
Cdc7-Dbf4 (Ddk)	Ddk
Cdc14	$Pase$

Variable	Symbol
Multisite phosphorylation of Sld2 and Sld3	
Free Sld2 with i phosphorylations	X_i
S-Cdk bound Sld2 with i phosphorylations	Xa_i
Cdc14 bound Sld2 with i phosphorylations	Xb_i
Free Sld3 with i phosphorylations	Y_i
S-Cdk bound Sld3 with i phosphorylations	Ya_i
Cdc14 bound Sld3 with i phosphorylations	Yb_i
Formation of 11-3-2 activator complex	
Dpb11	C_0
Dpb11-Sld2p	C_1
Sld3p-Dpb11	C_2
Sld3p-Dpb11-Sld2p	C_3

The model parameters and their reference values as well as the reference protein concentrations are listed in Supporting Table S1 and S2.

Activation of cell-cycle regulated kinases

In budding yeast, binding of DNA polymerases and initiation of DNA replication are controlled through phosphorylations by the cyclin-dependent kinases G1-Cdk (Cln1,2-Cdc28), S-Cdk (Clb5,6-Cdc28), and a third kinase Ddk (Dbf4-Cdc7).

The cell cycle begins with the synthesis of the initiator G1-cyclin Cln3, which binds to Cdc28 forming active Cln3-Cdk. Early in the cell cycle, the cyclin-dependent kinase inhibitor, Far1, blocks the activity of Cln3-Cdc28, that is continuously synthesized during growth (Alberghina et al, 2004). When the amount of Cln3-Cdk overcomes that of Far1, active Cln3-Cdk becomes free and, in turn, can phosphorylate the transcription factors SBF and MBF that become active. The transcriptional repressor of SBF, Whi5, is also phosphorylated by Cln3-Cdk and becomes translocated from the nucleus and thereby inactivated (Bloom & Cross, 2007). Among the targets of SBF/MBF are the genes that encode the G1-cyclins Cln1,2. Recent experiments suggest that G1-Cdk (Cln1,2-Cdc28) complexes can activate SBF/MBF and inhibit Whi5, creating a positive feedback loop (Skotheim et al, 2008). S-Cdk is activated by G1-Cdk that triggers the degradation of the S-Cdk inhibitor Sic1 (Verma et al, 1997; Nash et al, 2001). S-Cdk then acts at multiple targets including the proteins Sld2, Sld3, Mcm2-7 and ORCs to control the assembly of the pre-initiation complex and replication initiation (Nguyen et al, 2001; Tanaka et al, 2007; Zegerman & Diffley, 2007). A third kinase, Ddk, that also controls Mcm2-7 phosphorylation, is active before S-Cdk and appears to be less tightly regulated (Nougarede et al, 2000).

In the model we do not consider the mechanism of G1-Cdk activation explicitly. Instead we use the following sigmoid input-function to account for the positive feedback in G1-Cdk activation:

$$f_{Cln}(t) = \frac{1}{2}(\tanh[0.0075\ (t - 1500\ \text{s})] + 1)$$

We assumed that G1-Cdk becomes active 20 min after beginning of origin licensing, and Ddk activity is constant during G1 and S phase. The release of active S-Cdk following the multi-site phosphorylation and degradation of Sic1 is explained below.

Multisite phosphorylation of Sic1 and release of active S-Cdk

The S-Cdk inhibitor Sic1 is phosphorylated by G1-Cdk at at least six of nine Ser/Thr residues to bind to the SCF^{Cdc4} ubiquitin ligase (Nash et al, 2001; Deshaies & Ferrell, 2001; Orlicky et al, 2003), leading to the ubiquitination of Sic1, its degradation by the proteasome, and the release of active S-Cdk from inhibition. Mutation experiments suggest that it is the number of phosphorylated sites and not the identity of the individual sites that governs Sic1's ability to bind SCF^{Cdc4} (Nash et al, 2001).

Thus, we consider in the model random phosphorylation of nine phosphorylation sites in Sic1 by G1-Cdk. Phosphorylation reactions are counteracted by a phosphatase (likely to be Cdc14, as reported by Bloom & Cross, 2007; Visintin et al, 1998), which is assumed to dephosphorylate Sic1 randomly. Phosphorylation of any combination of at least six residues in Sic1 then leads to Sic1 degradation and the release of active S-Cdk. Details of the multi-step processes of ubiquitination and degradation of Sic1 are not considered in the model.

$$
\begin{aligned}
&\text{For } i = 0, ..., 5: \quad Sic_i\,'(t) = -k_{26}Cdk(t)Sic_i(t) + k_{-26}Z_i(t) \\
&\text{For } i = 6, ..., 9: \quad Sic_i\,'(t) = -k_{26}Cdk(t)Sic_i(t) + k_{-26}Z_i(t) - \delta_{27}Sic_i(t)
\end{aligned}
$$

$$
\begin{aligned}
Z_0\,'(t) &= k_{26}Cdk(t)Sic_0(t) - k_{-26}Z_0(t) - k_{24}Cln(t)f_{Cln}(t)Z_0(t) \\
&\quad + k_{-24}Za_0(t) + \beta_{25}Zb_1(t)
\end{aligned}
$$

For $i = 1, ..., 8$:

$$
\begin{aligned}
Z_i\,'(t) &= k_{26}Cdk(t)Sic_i(t) - k_{-26}Z_i(t) + (9-(i-1))\alpha_{24}Za_{i-1}(t) \\
&\quad - k_{25}Z_i(t)Pase(t) + k_{-25}Zb_i(t) - k_{24}Cln(t)f_{Cln}(t)Z_i(t) \\
&\quad + k_{-24}Za_i(t) + (i+1)\beta_{25}Zb_{i+1}(t) \\
Z_9\,'(t) &= k_{26}Cdk(t)Sic_9(t) - k_{-26}Z_9(t) + \alpha_{24}Za_8(t) \\
&\quad - k_{25}Z_9(t)Pase(t) + k_{-25}Zb_9(t)
\end{aligned}
$$

For $i = 0, ..., 8$:

$$
Za_i\,'(t) = k_{24}Cln(t)f_{Cln}(t)Z_i(t) - k_{-24}Za_i(t) - (9-i)\alpha_{24}Za_i(t)
$$

For $i = 1, ..., 9$:

$$
Zb_i\,'(t) = k_{25}Z_i(t)Pase(t) - k_{-25}Zb_i(t) - i\beta_{25}Zb_i(t)
$$

In the kinetic equations, i denotes the number of phosphorylated sites.

States of replication origins

The assembly of a replicative complex at each replication origin is a multi-step process. The model describes the sequence of origin modifications (transitions from S_0 to S_9) that eventually lead to origin firing and DNA replication. Several factors $(F_1, F_{2a}, F_{2b}, F_4, F_5)$ bind at each transition step. Some of these factors Cdc6 (F_1) and Cdt1 (F_{2a}) are only transiently recruited (steps 1, 3 and 9) (Randell et al, 2006), while Mcm2-7 (F_{2b}), Cdc45 (F_4) and GINS (F_5) are part of the replication machinery and travel with the replication fork (Gambus et al, 2006; Moyer et al, 2006; Pacek et al, 2006). The transition from the licensing to the firing phase (from state S_3 to S_4 in the model) depends on Mcm2-7 phosphorylations by two kinases, S-Cdk and Ddk (Nougarede et al, 2006; Sheu & Stillman, 2006). These phosphorylations allow Cdc45, that was so far only loosely associated with Mcm2-7, to become stably bound to Mcm molecules at the origins. We assumed that S-Cdk acts as a priming kinase (step 10) for the Mcm2-7 phosphorylation by Ddk (step 11) (Nougarede et al, 2000). The loading of GINS and DNA polymerase to the origins (transition from S_7 to S_8) are facilitated by the transiently recruited 11-3-2 activator (Sld3-Dpb11-Sld2, C_3) at the replication origins (Kanemaki & Labib, 2006). The exact sequence of protein recruitment at the origin is not clear. Alternative possibilities are discussed suggesting that Sld3 forms a complex with Cdc45 in the nucleoplasm, which can associate to the origins. Consequently, the complete 11-3-2 activator complex is formed on the DNA after phosphorylations of Sld3 and Sld2 by S-Cdk. This alternative mechanism would not interfere with the results of our mathematical model.

$$
\begin{aligned}
S_0\ '(t) &= -k_1 S_0(t) F_1(t) + k_{-1} S_1(t) - k_{23} S_0(t) Cdk(t) + k_{-23} Sc_0(t) \\
&\quad + k_{16} S_9(t) \\
S_1\ '(t) &= k_1 S_0(t) F_1(t) - k_{-1} S_1(t) - k_3 S_1(t) F_2(t) + k_{-3} S_2(t) \\
&\quad - k_{23} S_1(t) Cdk(t) + k_{-23} Sc_1(t) \\
S_2\ '(t) &= k_3 S_1(t) F_2(t) - k_{-3} S_2(t) - k_9 S_2(t) \\
S_3\ '(t) &= k_9 S_2(t) - S_3(t) k_{10} Cdk(t) + k_{-10} S_{Cdk}(t) - k_{23} S_3(t) Cdk(t) \\
&\quad + k_{-23} Sc_3(t) \\
S_{Cdk}\ '(t) &= S_3(t) k_{10} Cdk(t) - k_{-10} S_{Cdk}(t) - \alpha_{10} S_{Cdk}(t) \\
S_4\ '(t) &= \alpha_{10} S_{Cdk}(t) - S_4(t) k_{11} Ddk(t) + k_{-11} S_{Ddk}(t) \\
&\quad - k_{23} S_4(t) Cdk(t) + k_{-23} Sc_4(t) \\
S_{Ddk}\ '(t) &= k_{11} S_4(t) Ddk(t) - k_{-11} S_{Ddk}(t) - \alpha_{11} S_{Ddk}(t) \\
S_5\ '(t) &= \alpha_{11} S_{Ddk}(t) - k_{12} S_5(t) F_4(t) + k_{-12} S_6(t) - k_{23} S_5(t) Cdk(t) \\
&\quad + k_{-23} Sc_5(t) \\
S_6\ '(t) &= k_{12} S_5(t) F_4(t) - k_{-12} S_6(t) - k_{13} S_6(t) C_3(t) + k_{-13} S_7(t) \\
&\quad - k_{23} S_6(t) Cdk(t) + k_{-23} Sc_6(t) \\
S_7\ '(t) &= k_{13} S_6(t) C_3(t) - k_{-13} S_7(t) - k_{14} S_7(t) F_5(t) + k_{-14} S_8(t) \\
&\quad - k_{23} S_7(t) Cdk(t) + k_{-23} Sc_7(t) \\
S_8\ '(t) &= k_{14} S_7(t) F_5(t) - k_{-14} S_8(t) - k_{23} S_8(t) Cdk(t) + k_{-23} Sc_8(t) \\
&\quad - k_{15} S_8(t) \\
S_9\ '(t) &= k_{15} S_8(t) - k_{23} S_9(t) Cdk(t) + k_{-23} Sc_9(t) - k_{16} S_9(t)
\end{aligned}
$$

S-Cdk also inhibits the formation of the pre-replicative complex (S_3) by phosphorylating the ORC subunits Orc2 and Orc6 (Nguyen et al, 2001). ORC lacking Orc6 fails to interact with the licensing factor Cdt1 and to load the Mcm2-7 helicase onto the replication origin (Chen et al, 2007), raising the possibility that Orc6 phosphorylation inhibits Cdt1 binding (eventually for steric reasons). Thus, origins that are phosphorylated at ORC by S-Cdk before being licensed (in S_0 or S_1 states) cannot be licensed and, consequently, fired. However, origins that are phosphorylated at ORC after being licensed become protected against rereplication.

For $i = 0, 1, 3, 4, \ \dots\ , 9$:

$$
Sc_i\ '(t) = k_{23} S_i(t) Cdk(t) - k_{-23} Sc_i(t) - \alpha_{23} Sc_i(t)
$$

$$
\begin{aligned}
Sp_0\,'(t) &= -k_1 Sp_0(t)F_1(t) + k_{-1}Sp_1(t) + \alpha_{23}Sc_0(t) + k_{16}Sp_9(t) \\
Sp_1\,'(t) &= k_1 Sp_0(t)F_1(t) - k_{-1}Sp_1(t) + \alpha_{23}Sc_1(t) \\
Sp_3\,'(t) &= \alpha_{23}Sc_3(t) - Sp_3(t)k_{10}Cdk(t) + k_{-10}Sp_{Cdk}(t) \\
Sp_{Cdk}\,'(t) &= Sp_3(t)k_{10}Cdk(t) - k_{-10}Sp_{Cdk}(t) - \alpha_{10}Sp_{Cdk}(t) \\
Sp_4\,'(t) &= \alpha_{23}Sc_4(t) + \alpha_{10}Sp_{Cdk}(t) - Sp_4(t)k_{11}Ddk(t) \\
&\quad + k_{-11}Sp_{Ddk}(t) \\
Sp_{Ddk}\,'(t) &= k_{11}Sp_4(t)Ddk(t) - k_{-11}Sp_{Ddk}(t) - \alpha_{11}Sp_{Ddk}(t) \\
Sp_5\,'(t) &= \alpha_{23}Sc_5(t) + \alpha_{11}Sp_{Ddk}(t) - k_{12}Sp_5(t)F_4(t) + k_{-12}Sp_6(t) \\
Sp_6\,'(t) &= \alpha_{23}Sc_6(t) + k_{12}Sp_5(t)F_4(t) - k_{-12}Sp_6(t) - k_{13}Sp_6(t)C_3(t) \\
&\quad + k_{-13}Sp_7(t) \\
Sp_7\,'(t) &= \alpha_{23}Sc_7(t) + k_{13}Sp_6(t)C_3(t) - k_{-13}Sp_7(t) - k_{14}Sp_7(t)F_5(t) \\
&\quad + k_{-14}Sp_8(t) \\
Sp_8\,'(t) &= \alpha_{23}Sc_8(t) + k_{14}Sp_7(t)F_5(t) - k_{-14}Sp_8(t) - k_{15}Sp_8(t) \\
Sp_9\,'(t) &= \alpha_{23}Sc_9(t) + k_{15}Sp_8(t) - k_{16}Sp_9(t)
\end{aligned}
$$

In the model, we allow origins, that have already fired, to re-enter the initiation network (step 16) after a delay of ~ 8 minutes used for the ligation of DNA strands. These origins can then be licensed again (as long as the licensing molecules Cdc6 and Cdt1 are available and the inhibitory ORC phosphorylations by S-Cdk have not occurred), and finally progress to a second round of firing.
The number of rereplicating origins ρ is determined by subtracting the number of origins, that would fire when the re-entering of fired origins would not be allowed in the mathematical model ($k_{16} = 0$), from the calculated number of firing origins N, which also included the rereplicating origins.

Binding factors

Several proteins are required for the replication complex. The free concentration of these factors is reduced by binding to replication origins at particular states, by degradation and by export from the nucleus.

We assume that Cdt1 and Mcm2-7 form a stable complex (F_2) in the nucleus (step 4) and are recruited together to the replication origins (step 3). Mcm2-7, alone or in association with Cdt1, can be phosphorylated by G1-Cdk or S-Cdk (step 6) triggering its export from the nucleus (step 8) (Labib et al, 1999; Nguyen et al, 2000; Tanaka & Diffley, 2002). Free Cdt1 proteins also become excluded from the nucleus (step 7). After recruitment of Cdt1-Mcm2-7 to the origins, Cdc6 dissociates from the origins. G1-Cdk phosphorylates Cdc6, targeting it for degradation (step 2) (Drury et al, 2000).

$$
\begin{aligned}
F_1\,'(t) &= -k_1 F1(t)(S_0(t)+Sp_0(t)) + k_{-1}(S_1(t)+Sp_1(t)) + k_9 S_2(t) \\
&\quad -\delta_2 F_1(t) f_{Cln}(t) \\
F_{2a}\,'(t) &= -k_4 F_{2a}(t)F_{2b}(t) + k_{-4}F_2(t) - k_5 F_{2a}(t)F_{2bp}(t) + k_{-5}F_{2p}(t) \\
&\quad +k_9 S_2(t) - k_7 F_{2a}(t) \\
F_{2b}\,'(t) &= -k_4 F_{2a}(t)F_{2b}(t) + k_{-4}F_2(t) \\
&\quad -\alpha_6 F_{2b}(t)(f_{Cln}(t) + Cdk(t)/\mathrm{Clb56}) + k_{16}(S_9(t)+Sp_9(t)) \\
F_{2bp}\,'(t) &= \alpha_6 F_{2bp}(t)(f_{Cln}(t) + Cdk(t)/\mathrm{Clb56}) - k_5 F_{2a}(t)F_{2bp}(t) \\
&\quad +k_{-5}F_{2p}(t) - k_8 F_{2bp}(t) \\
F_2\,'(t) &= k_4 F_{2a}(t)F_{2b}(t) - k_{-4}F_2(t) - k_3 S_1(t)F_2(t) + k_{-3}S_2(t) \\
&\quad -\alpha_6 F_2(t)(f_{Cln}(t) + Cdk(t)/\mathrm{Clb56}) \\
F_{2p}\,'(t) &= \alpha_6 F_2(t)(f_{Cln}(t) + Cdk(t)/\mathrm{Clb56}) + k_5 F_{2a}(t)F_{2bp}(t) \\
&\quad -k_{-5}F_{2p}(t) - k_8 F_{2p}(t) \\
F_4\,'(t) &= -k_{12}F_4(t)(S_5(t)+Sp_5(t)) + k_{-12}(S_6(t)+Sp_6(t)) \\
&\quad +k_{16}(S_9(t)+Sp_9(t)) \\
F_5\,'(t) &= -k_{14}F_5(t)(S_7(t)+Sp_7(t)) + k_{-14}(S_8(t)+Sp_8(t)) \\
&\quad +k_{16}(S_9(t)+Sp_9(t))
\end{aligned}
$$

Multisite phosphorylation of Sld2 and Sld3

The S-Cdk target proteins Sld2 and Sld3 serve as key regulators that transiently associate with the replication origins and become dispensable after origin firing (Kanemaki & Labib, 2006). Phosphorylation of multiple serine and threonine residues in Sld2 and Sld3 is required for the binding to Dpb11 and the formation of an 11-3-2 activator complex that transiently associates with the replication origins and is critical for the recruitment of GINS, DNA polymerases and other factors to the replication origins (Tanaka et al, 2007; Zegerman & Diffley, 2007). The activation mechanism of Sld2 by S-Cdk is complex, requiring the phosphorylation of multiple Ser/Thr before the critical Thr84 site, the docking site for Dpb11, can be phosphorylated (Tak et al, 2006). Thr600 and Ser622 phosphorylations are required for Sld3 activation and binding to Dpb11 (Zegerman & Diffley, 2007). Sld2/Sld3 phosphorylation can be reversed by the phosphatase Cdc14, which exhibits, however, low activity levels during the S phase (Bloom & Cross, 2007).

According to *in vitro* measurements by Tak et al (2006), we considered in the model a random phosphorylation of six Ser/Thr residues ($X_i,\ i = 1,..,6$) in Sld2 followed by the Thr84 phosphorylation (X_7). For Sld3, random phosphorylation of two serines residues is assumed ($Y_i,\ i = 1,2$). Multisite phosphorylation of Sld2 and Sld3 is modeled as a distributive mechanism, in which the enzyme dissociates from the substrate after each modification and rebinds for the next step.

Although a random mechanism is generally more complicated due the large number of intermediate phosphorylation states (see Figure 6A of the main text), the system of differential equations can be reduced if we assume that the kinetic

parameters ($k_{17}, k_{-17}, \alpha_{17}, k_{18}, k_{-18}, \beta_{18}$) do not depend on the targets phosphorylation state (Salazar & Höfer, 2007). All i-times phosphorylated molecules can be then lumped into a single variable regardless of the position of the phosphorylated residues. In this way, the random mechanism is mapped to a chain of sequential reactions (see scheme below for Sld2), in which the phosphorylation rate decreases with increasing serine phosphorylation of the target with $(N-i+1)\alpha_{17}$, where N ist the total number of phosphorylation sites of the molecule. The dephosphorylation rate increases with $(i\beta_{18})$.

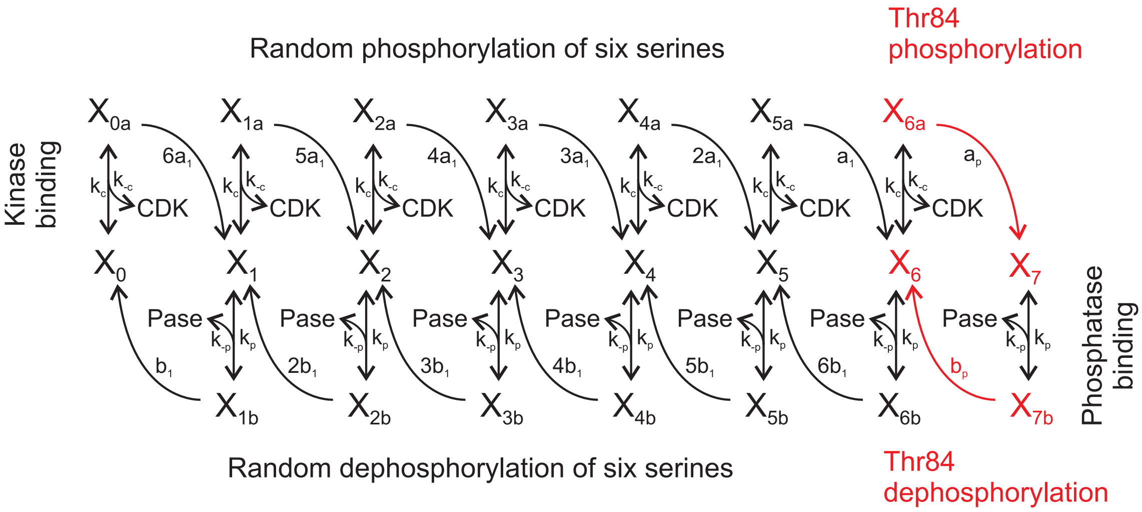

$$
\begin{aligned}
X_0\,'(t) &= -k_{17}X_0(t)Cdk(t) + k_{-17}Xa_0(t) + \beta_{18}Xb_1(t)\\
\text{For } i &= 1, ..., 5:\\
X_i\,'(t) &= (6-(i-1))\alpha_{17}Xa_0(t) - k_{17}X_1(t)Cdk(t) + k_{-17}Xa_1(t)\\
&\quad +(i+1)\beta_{18}Xb_2(t) - k_{18}X_1(t)Pase(t) + k_{-18}Xb_1(t)\\
X_6\,'(t) &= \alpha_{17}Xa_5(t) - k_{17}X_6(t)Cdk(t) + k_{-17}Xa_6(t) + \beta_{20}Xb_7(t)\\
&\quad -k_{18}X_6(t)Pase(t) + k_{-18}Xb_6(t)\\
X_7\,'(t) &= \alpha_{19}Xa_6(t) - k_{18}X_7(t)Pase(t) + k_{-18}Xb_7(t)\\
&\quad -k_{21}X_7(t)(C_0(t) + C_2(t)) + k_{-21}(C_1(t) + C_3(t))
\end{aligned}
$$

$$
\begin{aligned}
\text{For } i &= 0, 1, ..., 5:\\
Xa_i\,'(t) &= k_{17}X_i(t)Cdk(t) - k_{-17}Xa_i(t) - (6-i)\alpha_{17}Xa_i(t)\\
Xa_6\,'(t) &= k_{17}X_6(t)Cdk(t) - k_{-17}Xa_6(t) - \alpha_{19}Xa_6(t)
\end{aligned}
$$

$$
\begin{aligned}
\text{For } i &= 1, ..., 6:\\
Xb_i\,'(t) &= k_{18}X_i(t)Pase(t) - k_{-18}Xb_i(t) - i\beta_{18}Xb_i(t)\\
Xb_7\,'(t) &= k_{18}X_7(t)Pase(t) - k_{-18}Xb_7(t) - \beta_{20}Xb_7(t)
\end{aligned}
$$

$$\begin{aligned}
Y_0\,'(t) &= -k_{17}Y_0(t)Cdk(t) + k_{-17}Ya_0(t) + \beta_{18}Yb_1(t) \\
Y_1\,'(t) &= 2\alpha_{17}Ya_0(t) - k_{17}Y_1(t)Cdk(t) + k_{-17}Ya_1(t) + 2\beta_{18}Yb_2(t) \\
&\quad -k_{18}Y_1(t)Pase(t) + k_{-18}Yb_1(t) \\
Y_2\,'(t) &= \alpha_{17}Ya_1(t) - k_{18}Y_2(t)Pase(t) + k_{-18}Yb_2(t) \\
&\quad -k_{22}Y_2(t)(C_0(t) + C_1(t)) + k_{-22}(C_2(t) + C_3(t))
\end{aligned}$$

For $i = 0, 1$:

$$Ya_i\,'(t) = k_{17}Y_i(t)Cdk(t) - k_{-17}Ya_i(t) - (i+1)\alpha_{17}Ya_i(t)$$

For $i = 1, 2$:

$$Yb_i\,'(t) = k_{18}Y_i(t)Pase(t) - k_{-18}Yb_i(t) - \beta_{18}Yb_i(t)$$

Formation of the 11-3-2 catalytic complex

Multiple Thr/Ser phosphorylations in Sld2 and Sld3 are required for the formation of the 11-3-2 activator complex together with Dpb11. However, the sequence of the 11-3-2 activator assembly is presently not clear. Non-competitive protein interactions of phosphorylated Sld2 and Sld3 with Dpb11 may allow the pre-assembly of the 11-3-2 activator before binding to the replication origins (Tanaka et al, 2007; Zegerman & Difley, 2007). On the other hand, Yabuuchi et al (2006) have suggested, from experiments in fission yeast, the cooperative binding of Cdc45 and Sld3 to origins prior to recruitment of Sld2-Dpb11. It has been observed that 11-3-2 activator is no longer required after the replisome has started DNA synthesis. Specifically, Kanemaki & Labib (2006) suggested that the 11-3-2 activator is displaced form replication origins when the GINS complex binds.

In the model, we assume that fully phosphorylated Sld2 (X_7) and Sld3 (Y_2) can bind to Dpb11 (C_0) in a random order, allowing the pre-assembly of the 11-3-2 activator complex (C_3) before binding to the replication origins. The 11-3-2 complex binds to the replication origins in step 13 and dissociates from it in step 15 after the recruitment of the GINS complex and DNA polymerases.

$$\begin{aligned}
C_0\,'(t) &= -C_0(t)(k_{21}X_7(t) + k_{22}Y_2(t)) + k_{-21}C_1(t) + k_{-22}C_2(t) \\
C_1\,'(t) &= k_{21}X_7(t)C_0(t) - k_{22}Y_2(t)C_1(t) - k_{-21}C_1(t) + k_{-22}C_3(t) \\
C_2\,'(t) &= k_{22}Y_2(t)C_0(t) - k_{21}X_7(t)C_2(t) - k_{-22}C_2(t) + k_{-21}C_3(t) \\
C_3\,'(t) &= k_{22}Y_2(t)C_1(t) + k_{21}X_7(t)C_2(t) - k_{-22}C_3(t) - k_{-21}C_3(t) \\
&\quad -k_{13}C_3(t)(S_6(t) + Sp_6(t)) + k_{-13}(S_7(t) + Sp_7(t)) \\
&\quad +k_{15}(S_8(t) + Sp_8(t))
\end{aligned}$$

Cell-cycle dependent kinases and phosphatase

The free concentration of kinases S-Cdk and Ddk as well as of phosphatase Cdc14 (Pase) is given by the following kinetic equations:

$$
\begin{aligned}
Cln\ '(t) &= -k_{24}Cln(t)f_{Cln}(t)\sum_{i=0}^{8} Z_i(t) + k_{-24}\sum_{i=0}^{8} Za_i(t) \\
&+\alpha_{24}(\sum_{i=0}^{8}(9-i)Za_i(t))
\end{aligned}
$$

$$
\begin{aligned}
Cdk\ '(t) &= -k_{26}Cdk(t)\sum_{i=0}^{9} Sic_i(t) + k_{-26}\sum_{i=0}^{9} Z_i(t) \\
&-k_{17}Cdk(t)(\sum_{i=0}^{6} X_i(t) + \sum_{i=0}^{1} Y_i(t)) + k_{-17}(\sum_{i=0}^{6} Xa_i(t) + \sum_{i=0}^{1} Ya_i(t)) \\
&+\alpha_{17}(\sum_{i=0}^{5}(6-i)Xa_i(t) + \sum_{i=0}^{1}(2-i)Ya_i(t)) + \alpha_{19}Xa_6(t) \\
&-k_{23}Cdk(t)(\sum_{i=0}^{1} S_i(t) + \sum_{i=3}^{9} S_i(t)) + k_{-23}(\sum_{i=0}^{1} Sc_i(t) + \sum_{i=3}^{9} Sc_i(t)) \\
&+\alpha_{23}(\sum_{i=0}^{1} Sc_i(t) + \sum_{i=3}^{9} Sc_i(t)) - k_{10}Cdk(t)(S_3(t) + Sp_3(t)) \\
&+k_{-10}(S_{Cdk}(t) + Sp_{Cdk}(t)) + \alpha_{10}(S_{Cdk}(t) + Sp_{Cdk}(t))
\end{aligned}
$$

$$
\begin{aligned}
Pase\ '(t) &= -k_{25}Pase(t)\sum_{i=1}^{9} Z_i(t) + k_{-25}\sum_{i=1}^{9} Zb_i(t) + \beta_{25}(\sum_{i=1}^{9} iZb_i(t)) \\
&-k_{18}Pase(t)(\sum_{i=1}^{7} X_i(t) + \sum_{i=1}^{2} Y_i(t)) + k_{-18}(\sum_{i=1}^{7} Xb_i(t) + \sum_{i=1}^{2} Yb_i(t)) \\
&+\beta_{18}(\sum_{i=1}^{6} iXb_i(t) + \sum_{i=1}^{2} iYb_i(t)) + \beta_{20}Xb_7(t)
\end{aligned}
$$

$$
\begin{aligned}
Ddk\ '(t) &= -k_{11}Ddk(t)(S_4(t) + Sp_4(t)) + k_{-11}(S_{Ddk}(t) + Sp_{Ddk}(t)) \\
&+\alpha_{11}(S_{Ddk}(t) + Sp_{Ddk}(t))
\end{aligned}
$$

Initial conditions

We assume the following initial conditions: all origins are initially found at state S_0; Sld2 and Sld3 are completely dephosphorylated; all binding factors Cdc6, Cdt1, Mcm27, Cdc45, Sld2, Sld3, Dpb11 and GINS are free in the nucleus and not bound to other proteins. An initial concentration of zero is assumed for all other variables not listed below explicitly.

$$
\begin{aligned}
Cln(0) &= \text{Cln12}\\
Sic_0(0) &= \text{Sic1}\\
Cdk(0) &= \text{Clb56}\\
Pase(0) &= \text{Cdc14}\\
Ddk(0) &= \text{Cdc7}\\
S_0(0) &= \text{ORC}\\
F_1(0) &= \text{Cdc6}\\
F_{2a}(0) &= \text{Cdt1}\\
F_{2b}(0) &= \text{Mcm2-7}\\
F_4(0) &= \text{Cdc45}\\
F_5(0) &= \text{Gins}\\
X_0(0) &= \text{Sld2}\\
Y_0(0) &= \text{Sld3}\\
C_0(0) &= \text{Dpb11}
\end{aligned}
$$

References

Alberghina L, Rossi RL, Querin L, Wanke V, Vanoni M (2004) A cell sizer network involving Cln3 and Far1 controls entrance into S phase in the mitotic cycle of budding yeast. *J Cell Biol* **167**: 433-443

Bloom J, Cross FR (2007) Novel role for Cdc14 sequestration: Cdc14 dephosphorylates factors that promote DNA replication. *Mol Cell Biol* **27**: 842-853

Chen S, de Vries MA, Bell SP (2007) Orc6 is required for dynamic recruitment of Cdt1 during repeated Mcm2-7 loading. *Genes Dev* **21**: 2897-2907

Deshaies RJ, Ferrell JE Jr (2001) Multisite phosphorylation and the countdown to S phase. *Cell* **107**, 819-822

Drury LS, Perkins G, Diffley JF (2000) The cyclin-dependent kinase Cdc28p regulates distinct modes of Cdc6p proteolysis during the budding yeast cell cycle. *Curr Biol* **10**: 231-240

Gambus A, Jones RC, Sanchez-Diaz A, Kanemaki M, van Deursen F, Edmondson RD, Labib K (2006) GINS maintains association of Cdc45 with MCM in replisome progression complexes at eukaryotic DNA replication forks. *Nat Cell Biol* **8(4)**: 358-366

Kanemaki M, Labib K (2006) Distinct roles for Sld3 and GINS during establishment and progression of eukaryotic DNA replication forks. *EMBO J* **25**: 1753-1763

Labib K, Diffley JF, Kearsey SE (1999) G1-phase and B-type cyclins exclude the DNA-replication factor Mcm4 from the nucleus. *Nat Cell Biol* **1(7)**: 415-422

Moyer SE, Lewis PW, Botchan MR (2006) Isolation of the Cdc45/Mcm2-7/GINS (CMG) complex, a candidate for the eukaryotic DNA replication fork helicase. *Proc Natl Acad Sci USA* **103**: 10236-10241

Nash P, Tang X, Orlicky S, Chen Q, Gertler FB, Mendenhall MD, Sicheri F, Pawson T, Tyers M (2001) Multisite phosphorylation of a CDK inhibitor sets a threshold for the onset of DNA replication. *Nature* **414**: 514-521

Nguyen VQ, Co C, Irie K, Li JJ (2000) Clb/Cdc28 kinase promote nuclear export of the replication initiator proteins Mcm2-7. *Curr Biol* **10(4)**: 195-205

Nguyen VQ, Co C, Li JJ (2001) Cyclin-dependent kinases prevent DNA re-replication through multiple mechanisms. *Nature* **411**: 1068-1073

Nougarede R, Della Seta F, Zarzov P, Schwob E (2000) Hierarchy of S-phase-promoting factors: yeast Dbf4-Cdc7 kinase requires prior S-phase cyclin-dependent kinase activation. *Mol Cell Biol* **20**: 3795-3806

Orlicky S, Tang X, Willems A, Tyers M, Sicheri F. (2003) Structural basis for phosphodependent substrate selection and orientation by the SCFCdc4 ubiquitin ligase. *Cell* **112**, 243-256

Pacek M, Tutter AV, Kubota Y, Takisawa H, Walter JC (2006) Localization of MCM2-7, Cdc45, and GINS to the site of DNA unwinding during eukaryotic DNA replication. *Mol Cell* **21**: 581-587

Randell JC, Bowers JL, Rodriguez HK, Bell SP (2006) Sequential ATP hydrolysis by Cdc6 and ORC directs loading of the Mcm2-7 helicase. *Mol Cell* **6**: 29-39

Salazar C, Höfer T (2007) Versatile regulation of multisite protein phosphorylation by the order of phosphate processing and protein-protein interactions. *FEBS J* **274**: 1046-1061

Sheu YJ, Stillman B (2006) Cdc7-Dbf4 phosphorylates MCM proteins via a docking site-mediated mechanism to promote S phase progression. *Mol Cell* **24**: 101-113

Skotheim JM, Di Talia S, Siggia ED, Cross FR (2008) Positive feedback of G1 cyclins ensures coherent cell cycle entry. *Nature* **454**: 291-296

Tak YS, Tanaka Y, Endo S, Kamimura Y, Araki H (2006) A CDK-catalysed regulatory phosphorylation for formation of the DNA replication complex Sld2-Dpb11. *EMBO J* **25**: 1987-1996

Tanaka S, Diffley JF (2002) Interdependent nuclear accumulation of budding yeast Cdt1 and Mcm2-7 during G1 phase. *Nat Cell Biol* **4(3)**: 198-207

Tanaka S, Umemori T, Hirai K, Muramatsu S, Kamimura Y, Araki H (2007) CDK-dependent phosphorylation of Sld2 and Sld3 initiates DNA replication in budding yeast. *Nature* **445**: 328-332

Verma R, Annan RS, Huddleston MJ, Carr SA, Reynard G, Deshaies RJ (1997) Phosphorylation of Sic1p by G1-Cdk required for its degradation and entry into S phase. *Science* **278**: 455-460

Visintin R, Craig K, Hwang ES, Prinz S, Tyers M, Amon A (1998) The phosphatase Cdc14 triggers mitotic exit by reversal of Cdk-dependent phosphorylation. *Mol Cell* **2**: 709-718

Yabuuchi H, Yamada Y, Uchida T, Sunathvanichkul T, Nakagawa T, Masukata H (2006) Ordered assembly of Sld3, GINS and Cdc45 is distinctly regulated by DDK and CDK for activation of replication origins. *EMBO J* **25:** 4663-4674

Zegerman P, Diffley JF (2007) Phosphorylation of Sld2 and Sld3 by cyclin-dependent kinases promotes DNA replication in budding yeast. *Nature* **445**: 281-285
