# Supplementary material for: Mathematical Modelling of DNA Replication Reveals a Trade-off between Coherence of Origin Activation and Robustness against Rereplication
Source: PLoS Comput Biol. 2010 May 13;6(5):e1000783. doi: 10.1371/journal.pcbi.1000783 (PMC2869307; doi:10.1371/journal.pcbi.1000783)
Supplement: Text S2 — Determination of kinetic parameter values (0.04 MB PDF) [file pcbi.1000783.s002.pdf]

Supporting Text 2: Determination of kinetic parameter values

The parameters of the model can be grouped into three categories: protein concentrations, binding/dissociation rate constants, and rate constants of phosphorylation/dephosphorylation. Since most of the model parameters has not been determined experimentally, we parametrized the molecular network by asking for which sets of parameters the network is functional in terms of (1) ensuring efficient and coherent firing of the replication origins and (2) preventing rereplication. Optimized parameter sets have to fulfill the following conditions:

(i) Minimal duration of origin firing Δ.

(ii) Total fraction of firing origins above 95%.

(iii) Number of rereplicating origins per cell-cycle below 0.01.

We fixed the protein concentrations (Supporting Table 1), and the kinetic parameters of the licensing and S-Cdk-activation module (Supporting Table 2) according to experimental data (Ghaemmaghami et al, 2003). The kinetic parameters of the 11-3-2 activator and firing module (Supporting Table 2) were optimized within biochemically reasonable ranges:

$$\begin{aligned}
\text{binding rate constants } k_{on} &\in [10^3, \ \dots, 10^6] \text{ M}^{-1}\text{s}^{-1} \\
\text{equilibrium dissociation constants } K_\text{d} &\in [10^{-8}, \ \dots, 10^{-5}] \text{ M} \\
\text{phosphorylation rate constants } \alpha &\in [0.01, \ \dots, 1] \text{ s}^{-1} \\
\text{dephosphorylation rate constants } \beta &\in [0.05, \ \dots, 5] \text{ s}^{-1}
\end{aligned}$$

For binding rate constants of protein-protein and protein-DNA interactions, a range of values somewhat below the theoretical difussion limit was chosen, off-rate constants were adjusted as to obtain characteristic micromolar to nanomolar dissociation constants (Gabdoulline & Wade, 2001). Measured reaction times for protein phosphorylation and dephosphorylarion are usually in the second-to-subsecond range, with phospho-transfer reactions being somewhat slower than cleavage of phosphate groups (e.g. Shaw et al, 1995; Okamura et al, 2004).

For several protein binding steps in the sequence of replication complex assembly, dissociation reactions were quasi neglected under the assumption that the subsequent step occurs more rapidly than dissociation. Thus, five reactions (steps 12, 13, 14, 21 and 22) were assumed to be almost irreversible and the dissociation rates $k_{-12}, k_{-13}, k_{-14}, k_{-21}$ and k_{-22} were fixed to 0.005 s^{-1} to facilitate the optimization procedure. The model parameters were optimized on a logarithmic scale within these ranges to allow parameter values that are equally distributed over the allowed orders of magnitude. The optimization procedure was implemented in Mathematica 7.0 using the "NMinimize" function and the "DifferentialEvolution" method. The optimization routine was run 184 times using different seeds in the random number generator and fixing the number of iterations in order to limit the time spent for the

search of a single parameter set. The program was not able to identify an optimized parameter set in each run, instead we obtained 109 admissible parameter sets that fulfill our criteria but show somewhat different firing kinetics with respect to the degree of coherence and the mean firing time. The distribution of kinetic parameters in the admissible sets is shown in Supporting Figures 1 and 2 and the mean values and standard deviations of all optimized parameter sets are listed in Supporting Table 3.

In Figure 4A and B of the main text, the initial protein concentrations listed in Supporting Table 1 and the kinetic parameters listed Supporting Table 2 were used in to simulate the initiation of DNA replication under normal conditions. The situation with a slow degradation of Sic1 (Figure 4C and D of the main text) was realized by a 100-fold reduction of the degradation rate of Sic1 (δ_{27}) to 0.01 1/sec. To reflect the conditions in the ΔSic1 mutant (Figure 4E and F of the main text), the initial concentration of Sic1 was set to zero and at the same time the initial concentration of S-Cdk was halved corresponding to 300 molecules per nucleus. The situation in experiments by Zegerman & Diffley (2007) and Tanaka et al (2007) (Figure 4G and H of the main text) was realized by setting the degradation rate of Sic1 (δ_{27}) to zero and additionally introducing an initial number of 200 molecules of the 11-3-2 activator complex (variable $C3$ in the differential equations) into the mathematical model.

References

Gabdoulline RR, Wade RC (2001) Protein-protein association: investigation of factors influencing association rates by brownian dynamics simulations. *J Mol Biol* **306(5)**: 1139-1155

Okamura H, Garcia-Rodriguez C, Martinson H, Qin J, Virshup DM, Rao A (2004) A conserved motif for CK1 binding controls the nuclear localization of NFAT1. *Mol Cell Biol* **24(10)**: 4184-4195

Shaw AS, Gauen LK, Zhu Y (1995) Interactions of TCR tyrosine based activation motifs with tyrosine kinases. *Semin Immunol* **7(1)**: 13-20

Tanaka S, Umemori T, Hirai K, Muramatsu S, Kamimura Y, Araki H (2007) CDK-dependent phosphorylation of Sld2 and Sld3 initiates DNA replication in budding yeast. *Nature* **445**: 328-332

Zegerman P, Diffley JF (2007) Phosphorylation of Sld2 and Sld3 by cyclin-dependent kinases promotes DNA replication in budding yeast. *Nature* **445**: 281-285
