# Supplementary material for: Mathematical Modelling of DNA Replication Reveals a Trade-off between Coherence of Origin Activation and Robustness against Rereplication
Source: PLoS Comput Biol. 2010 May 13;6(5):e1000783. doi: 10.1371/journal.pcbi.1000783 (PMC2869307; doi:10.1371/journal.pcbi.1000783)
Supplement: Text S3 — Calculation of replicon sizes (0.05 MB PDF) [file pcbi.1000783.s003.pdf]

Supporting Text 3: Calculation of replicon sizes

To quantify the consequences of un-/synchronous origin activation on the DNA synthesis period, we calculated the length and the completion time of each replicon for all firing origins. The positions of the 190 replication-origins $x_0(i)$ on DNA were assigned using the experimentally measured distribution of inter-origin distances in budding yeast (Lengronne et al, 2001). A firing time $t_0(i)$ is randomly selected out of the distribution of firing times derived from the model (see Figure 1B of the main text) for each origin i at position $x(i)$. DNA is then assumed to be synthesized bidirectionally with the same synthesis rate v= 48.8 bp/s (Raghuraman et al, 2001) by polymerases, starting in both directions simultaneously at each replication origin. One replicon $R(i)$ of an origin i is defined as the total piece of DNA that is synthesized by these polymerases in 3' and 5' direction. The replicon size $R(i)$ depends on the differences in firing times, $\Delta t_0(i) = t_0(i) - t_0(i-1)$ and $\Delta t_0(i+1) = t_0(i+1) - t_0(i)$, and the distances, $\Delta x_0(i) = x_0(i) - x_0(i-1)$ and $\Delta x_0(i+1) = x_0(i+1) - x_0(i)$, between the origin i and both of its neighboring origins, $i-1$ and $i+1$. The replicon ends were defined by the points where two replication forks that move in opposing directions collapse (Labib & Hodgson, 2007).

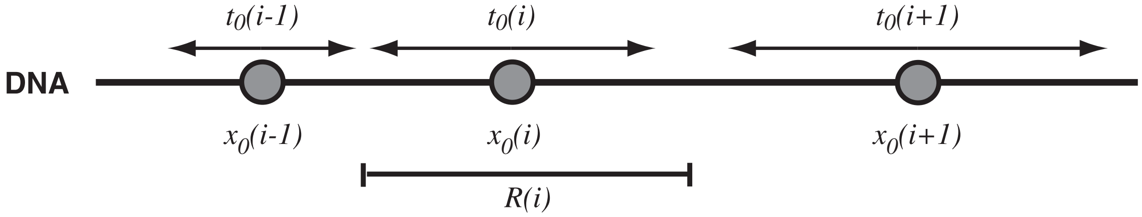

The replicon size $R(i)$ of an origin i can be calculated by the following formula:

$$R(i) = R_l(i) + R_r(i)$$

with:

$$R_l(i) = \begin{cases} \Delta x_0(i) & \text{if } v\Delta t_0(i) \geq \Delta x_0(i) \text{ and } t_0(i) > t_0(i-1) \\ 0 & \text{if } v\Delta t_0(i) \geq \Delta x_0(i) \text{ and } t_0(i) < t_0(i-1) \\ \frac{\Delta x_0(i)}{2} + \frac{v\Delta t_0(i)}{2} & \text{if } v\Delta t_0(i) < \Delta x_0(i) \text{ and } t_0(i) \geq t_0(i-1) \\ \frac{\Delta x_0(i)}{2} - \frac{v\Delta t_0(i)}{2} & \text{if } v\Delta t_0(i) < \Delta x_0(i) \text{ and } t_0(i) > t_0(i-1) \end{cases}$$

and analog:

$$R_r(i) = \begin{cases} 0 & \text{if } v\Delta t_0(i+1) \geq \Delta x_0(i+1) \text{ and } t_0(i+1) > t_0(i) \\ \Delta x_0(i+1) & \text{if } v\Delta t_0(i+1) \geq \Delta x_0(i+1) \text{ and } t_0(i+1) < t_0(i) \\ \frac{\Delta x_0(i+1)}{2} - \frac{v\Delta t_0(i+1)}{2} & \text{if } v\Delta t_0(i+1) < \Delta x_0(i+1) \text{ and } t_0(i+1) \geq t_0(i) \\ \frac{\Delta x_0(i+1)}{2} + \frac{v\Delta t_0(i+1)}{2} & \text{if } v\Delta t_0(i+1) < \Delta x_0(i+1) \text{ and } t_0(i+1) > t_0(i) \end{cases}$$

The time between the first origin firing event and the completion of the last DNA replicon is taken to be the duration of the early S phase. The duration of the early S phase is calculated under the assumption that the replication forks move bidirectionally with the same speed, v= 48.8 bp/s = 2.9 kbp/min (Raghuraman et al, 2001). If a faster progression speed of replication fork would be used, e.g. v= 61.7 bp/s = 3.7 kbp/min (Lengronne et al, 2001), the calculated duration of the early S phase would be shorter. Using the reference parameter set under normal conditions, the duration of the early S phase would then be shortened by around four minutes and take 23.5 minutes instead of 27.4 minutes.
The distribution of replicon sizes as well as the duration of the early S phase is averaged over 100 calculations with stochastically assigned the distances and firing times to the origins in each case according to the measured distribution of inter-origin-distances (Lengronne et al, 2001) and the calculated firing rate $f(t)$.

References

Labib K, Hodgson B (2007) Replication fork barriers: pausing for a break or stalling for time. *EMBO Rep* **8(4)**: 346-353

Lengronne A, Pasero P, Bensimon A, Schwob E (2001) Monitoring S phase progression globally and locally using BrdU incorporation in TK(+) yeast strains. *Nucleic Acids Res* **29**: 1433-1442

Raghuraman MK, Winzeler EA, Collingwood D, Hunt S, Wodicka L, Conway A, Lockhart DJ, Davis RW, Brewer BJ, Fangman WL (2001) Replication dynamics of the yeast genome. *Science* **294**: 115-121
