# Supplementary material for: Mathematical Modelling of DNA Replication Reveals a Trade-off between Coherence of Origin Activation and Robustness against Rereplication
Source: PLoS Comput Biol. 2010 May 13;6(5):e1000783. doi: 10.1371/journal.pcbi.1000783 (PMC2869307; doi:10.1371/journal.pcbi.1000783)
Supplement: Table S1 — Protein numbers and concentrations (0.03 MB PDF) [file pcbi.1000783.s004.pdf]

Supporting Table 1: Protein numbers and concentrations

Protein	Value	Unit	Reference/Remarks
Cdc6	700, 233	#, nM	
Cdt1	2200, 733	#, nM	*a*
Mcm2-7	2000, 667	#, nM	*a*
Sld2	660, 220	#, nM	*a*
Sld3	130, 43	#, nM	*a*
Dpb11	540, 180	#, nM	*a*
Cln1,2	1500, 500	#, nM	*a*
Sic1	800, 267	#, nM	*a*
Clb5,6	600, 200	#, nM	*a* ([Clb5] =520); *b* ($\frac{[\text{Clb6}]}{[\text{Clb5}]} \approx 10\%$)
Cdc7	1600, 533	#, nM	*a*
Cdc14	360, 120	#, nM	*a*
Cdc45	1700, 567	#, nM	*a*
GINS	1500, 500	#, nM	*a*
Early origins	190	#	*c*
Volume of the nucleus	5×10^{-15}	l	*d*

[a]Ghaemmaghami et al (2003)
[b]Cross et al (2002)
[c]Lengronne et al (2001)
[d]Jorgensen et al (2007)

References

Cross FR, Archambault V, Miller M, Klovstad M (2002) Testing a mathematical model of the yeast cell cycle. *Mol Biol Cell* **13**: 52-70

Ghaemmaghami S, Huh WK, Bower K, Howson RW, Belle A, Dephoure N, O'Shea EK, Weissman JS (2003) Global analysis of protein expression in yeast. *Nature* **425**: 737-741

Jorgensen P, Edgington NP, Schneider BL, Rupes I, Tyers M, Futcher B (2007) The size of the nucleus increases as yeast cells grow. *Mol Biol Cell* **18**: 3523-3532

Lengronne A, Pasero P, Bensimon A, Schwob E (2001) Monitoring S phase progression globally and locally using BrdU incorporation in TK(+) yeast strains. *Nucleic Acids Res* **29**: 1433-1442
