# Supplementary material for: Mathematical Modelling of DNA Replication Reveals a Trade-off between Coherence of Origin Activation and Robustness against Rereplication
Source: PLoS Comput Biol. 2010 May 13;6(5):e1000783. doi: 10.1371/journal.pcbi.1000783 (PMC2869307; doi:10.1371/journal.pcbi.1000783)
Supplement: Table S2 — Kinetic parameters of the mathematical model (0.04 MB PDF) [file pcbi.1000783.s005.pdf]

Supporting Table 2: Kinetic parameters of the mathematical model

Parameter	Symbol	Value	Unit	
Licensing				
Binding of Cdc6	k_1	2×10^5	$(\mathrm{Ms})^{-1}$	[a,b]
Dissociation of Cdc6	k_{-1}	0.2	s^{-1}	[a,b]
Degradation of Cdc6	δ_2	0.005	s^{-1}	[a,c]
Binding of Cdt1-Mcm2-7	k_3	1×10^5	$(\mathrm{Ms})^{-1}$	[a]
Dissociation of Cdt1-Mcm2-7	k_{-3}	0.05	s^{-1}	[a]
Binding of Cdt1 and Mcm2-7	k_4	2×10^5	$(\mathrm{Ms})^{-1}$	[a]
Dissociation of Cdt1 and Mcm2-7	k_{-4}	0.02	s^{-1}	[a]
Binding of Cdt1 and Mcm2-7p	k_5	2×10^3	$(\mathrm{Ms})^{-1}$	[a]
Dissociation of Cdt1 and Mcm2-7p	k_{-5}	2	s^{-1}	[a]
Phosphorylation of (Cdt1-)Mcm2-7	α_6	0.002	s^{-1}	[a]
Export of Cdt1	k_7	0.005	s^{-1}	[a]
Export of (Cdt1-)Mcm2-7p	k_8	0.005	s^{-1}	[a]
Dissociation of Cdc6, Cdt1	k_9	0.167	s^{-1}	[a]
S-Cdk activation				
Binding of G_1-Cdk and S-Cdk-Sic1	k_{24}	5×10^5	$(\mathrm{Ms})^{-1}$	[a]
Dissociation of G_1-Cdk and S-Cdk-Sic1	k_{-24}	5	s^{-1}	[a]
Phosphorylation of S-Cdk-Sic1	α_{24}	0.1	s^{-1}	[a]
Binding of Cdc14 and S-Cdk-Sic1	k_{25}	5×10^4	$(\mathrm{Ms})^{-1}$	[a]
Dissociation of Cdc14 and S-Cdk-Sic1	k_{-25}	0.5	s^{-1}	[a]
Dephosphorylation of S-Cdk-Sic1	β_{25}	2.5	s^{-1}	[a]
Binding of Sic1(ip) and S-Cdk	k_{26}	2.5×10^6	$(\mathrm{Ms})^{-1}$	[a,d]
Dissociation of Sic1(ip) and S-Cdk	k_{-26}	0.0092	s^{-1}	[a,e]
Degradation of Sic1(ip), $i = 6, .., 9$	δ_{27}	1	s^{-1}	[a]

[a] Fixed, not optimized parameter value
[b] Feng et al (2000)
[c] Drury et al (2000)
[d] Barberis et al (2007)
[e] Barberis et al (2005)

11-3-2 activator formation				
Binding of S-Cdk and Sld2/3	k_{17}/k_{19}	6.4×10^4	$(\mathrm{Ms})^{-1}$	*f*
Dissociation of S-Cdk and Sld2/3	k_{-17}/k_{-19}	7.9×10^{-3}	s^{-1}	*f*
Phosphorylation of Sld2/3	α_{17}	0.2	s^{-1}	*f*
Phosphorylation of Thr84 in Sld2	α_{19}	0.9	s^{-1}	*f*
Binding of Cdc14 and Sld2/3	k_{18}/k_{20}	1.1×10^3	$(\mathrm{Ms})^{-1}$	*f*
Dissociation of Cdc14 and Sld2/3	k_{-18}/k_{-20}	9.9×10^{-3}	s^{-1}	*f*
Dephosphorylation of Sld2/3	β_{18}	0.3	s^{-1}	*f*
Dephosphorylation of Thr84 in Sld2	β_{20}	0.5	s^{-1}	*f*
Binding of Sld2p and Dpb11	k_{21}	5.0×10^5	$(\mathrm{Ms})^{-1}$	*f*
Dissociation of Sld2p and Dpb11	k_{-21}	0.005	s^{-1}	*a*
Binding of Sld3p and Dpb11	k_{22}	1.7×10^5	$(\mathrm{Ms})^{-1}$	*f*
Dissociation of Sld3p and Dpb11	k_{-22}	0.005	s^{-1}	*a*
Firing				
Binding of S-Cdk and Mcm2-7	k_{10}	7.3×10^4	$(\mathrm{Ms})^{-1}$	*f*
Dissociation of S-Cdk and Mcm2-7	k_{-10}	8.2×10^{-3}	s^{-1}	*f*
Phosphorylation of Mcm2-7 by S-Cdk	α_{10}	0.1	s^{-1}	*f*
Binding of Ddk and Mcm2-7	k_{11}	3.0×10^4	$(\mathrm{Ms})^{-1}$	*f*
Dissociation of Ddk and Mcm2-7	k_{-11}	0.01	s^{-1}	*f*
Phosphorylation of Mcm2-7 by Ddk	α_{11}	0.8	s^{-1}	*f*
Binding of Cdc45	k_{12}	1.1×10^5	$(\mathrm{Ms})^{-1}$	*f*
Dissociation of Cdc45	k_{-12}	0.005	s^{-1}	*b*
Binding of Sld3p-Dpb11-Sld2p	k_{13}	5×10^5	$(\mathrm{Ms})^{-1}$	*f*
Dissociation of Sld3p-Dpb11-Sld2p	k_{-13}	0.005	s^{-1}	*a*
Binding of GINS and polymerase	k_{14}	3.3×10^5	$(\mathrm{Ms})^{-1}$	*f*
Dissociation of GINS and polymerase	k_{-14}	0.005	s^{-1}	*a*
Dissociation of 11-3-2 activator	k_{15}	0.9	s^{-1}	*f*
Dissociation of Mcm2-7, Cdc45, GINS, and polymerase; resetting of origins after replicon completion	k_{16}	0.005	s^{-1}	*a*
Binding of S-Cdk and Orc6	k_{23}	6.3×10^3	$(\mathrm{Ms})^{-1}$	*f*
Dissociation of S-Cdk and Orc6	k_{-23}	9.2×10^{-3}	s^{-1}	*f*
Phosphorylation of Orc6	α_{23}	0.7	s^{-1}	*f*

[a]Fixed, not optimized parameter value
[f]Parameter value from a reference set, selected from all 109 optimized parameter sets

References

Barberis M, Klipp E, Vanoni M, Alberghina L (2007) Cell size at S phase initiation: an emergent property of the G1/S network. *PLoS Comput Biol* **3**: e64

Barberis M, De Gioia L, Ruzzene M, Sarno S, Coccetti P, Fantucci P, Vanoni M, Albergina L (2005) The yeast cyclin-dependent kinase inhibitor Sic1 and mammalian p27Kip1 are functional homologues with a structurally conserved inhibitory domain. *Biochem J* **387**: 639-647

Drury LS, Perkins G, Diffley JF (2000) The cyclin-dependent kinase Cdc28p regulates distinct modes of Cdc6p proteolysis during the budding yeast cell cycle. *Curr Biol* **10**: 231-240

Feng L, Wang B, Driscoll B, Jong A (2000) Identification and characterization of Saccharomyces cerevisiae Cdc6 DNA-binding properties. *Mol Biol Cell* **11**: 1673-1685
