# Supplementary material for: Mathematical Modelling of DNA Replication Reveals a Trade-off between Coherence of Origin Activation and Robustness against Rereplication
Source: PLoS Comput Biol. 2010 May 13;6(5):e1000783. doi: 10.1371/journal.pcbi.1000783 (PMC2869307; doi:10.1371/journal.pcbi.1000783)
Supplement: Table S3 — Mean value and standard deviation of optimized parameters (0.02 MB PDF) [file pcbi.1000783.s006.pdf]

Supporting Table 3: Mean value and standard deviation of optimized parameters

Parameter	Unit	Mean value	Standard deviation	rel. Std
k_{10}	$(\mathrm{Ms})^{-1}$	7.19×10^{4}	2.66×10^{4}	0.37
Kd_{10}	M	2.86×10^{-7}	9.50×10^{-7}	3.32
α_{10}	s^{-1}	0.24	0.31	1.26
k_{11}	$(\mathrm{Ms})^{-1}$	3.80×10^{4}	2.95×10^{4}	0.78
Kd_{11}	M	8.80×10^{-7}	1.57×10^{-6}	1.78
α_{11}	s^{-1}	0.31	0.34	1.08
k_{12}	$(\mathrm{Ms})^{-1}$	2.27×10^{5}	1.69×10^{5}	0.74
Kd_{12}	M	5.14×10^{-8}	6.34×10^{-8}	1.23
k_{13}	$(\mathrm{Ms})^{-1}$	4.65×10^{5}	8.87×10^{-4}	0.19
Kd_{13}	M	1.21×10^{-8}	8.43×10^{-9}	0.70
k_{14}	$(\mathrm{Ms})^{-1}$	2.44×10^{5}	1.59×10^{5}	0.65
Kd_{14}	M	4.28×10^{-8}	5.18×10^{-8}	1.21
k_{15}	s^{-1}	0.27	0.29	1.10
k_{17}/k_{19}	$(\mathrm{Ms})^{-1}$	7.58×10^{4}	2.88×10^{4}	0.38
Kd_{17}/Kd_{19}	M	2.53×10^{-7}	3.52×10^{-7}	1.39
α_{17}	s^{-1}	0.31	0.33	1.07
α_{19}	s^{-1}	0.28	0.33	1.17
k_{18}/k_{20}	$(\mathrm{Ms})^{-1}$	7.07×10^{3}	7.41×10^{3}	1.05
Kd_{18}/Kd_{20}	M	3.36×10^{-6}	3.02×10^{-6}	0.90
β_{18}	s^{-1}	1.24	1.55	1.24
β_{20}	s^{-1}	1.50	1.48	0.98
k_{21}	$(\mathrm{Ms})^{-1}$	2.70×10^{5}	1.70×10^{5}	0.63
Kd_{21}	M	3.65×10^{-8}	3.87×10^{-8}	1.06
k_{22}	$(\mathrm{Ms})^{-1}$	2.56×10^{5}	1.74×10^{5}	0.68
Kd_{22}	M	4.89×10^{-8}	7.60×10^{-8}	1.56
k_{23}	$(\mathrm{Ms})^{-1}$	1.15×10^{4}	4.94×10^{3}	0.43
Kd_{23}	M	1.18×10^{-6}	1.48×10^{-6}	1.26
α_{23}	s^{-1}	0.44	0.36	0.83
