# Supplementary figures and images for: Mathematical Modelling of DNA Replication Reveals a Trade-off between Coherence of Origin Activation and Robustness against Rereplication
Source: PLoS Comput Biol. 2010 May 13;6(5):e1000783. doi: 10.1371/journal.pcbi.1000783 (PMC2869307; doi:10.1371/journal.pcbi.1000783)

# Supporting Figure 1: Distribution of optimized parameter values of the firing module

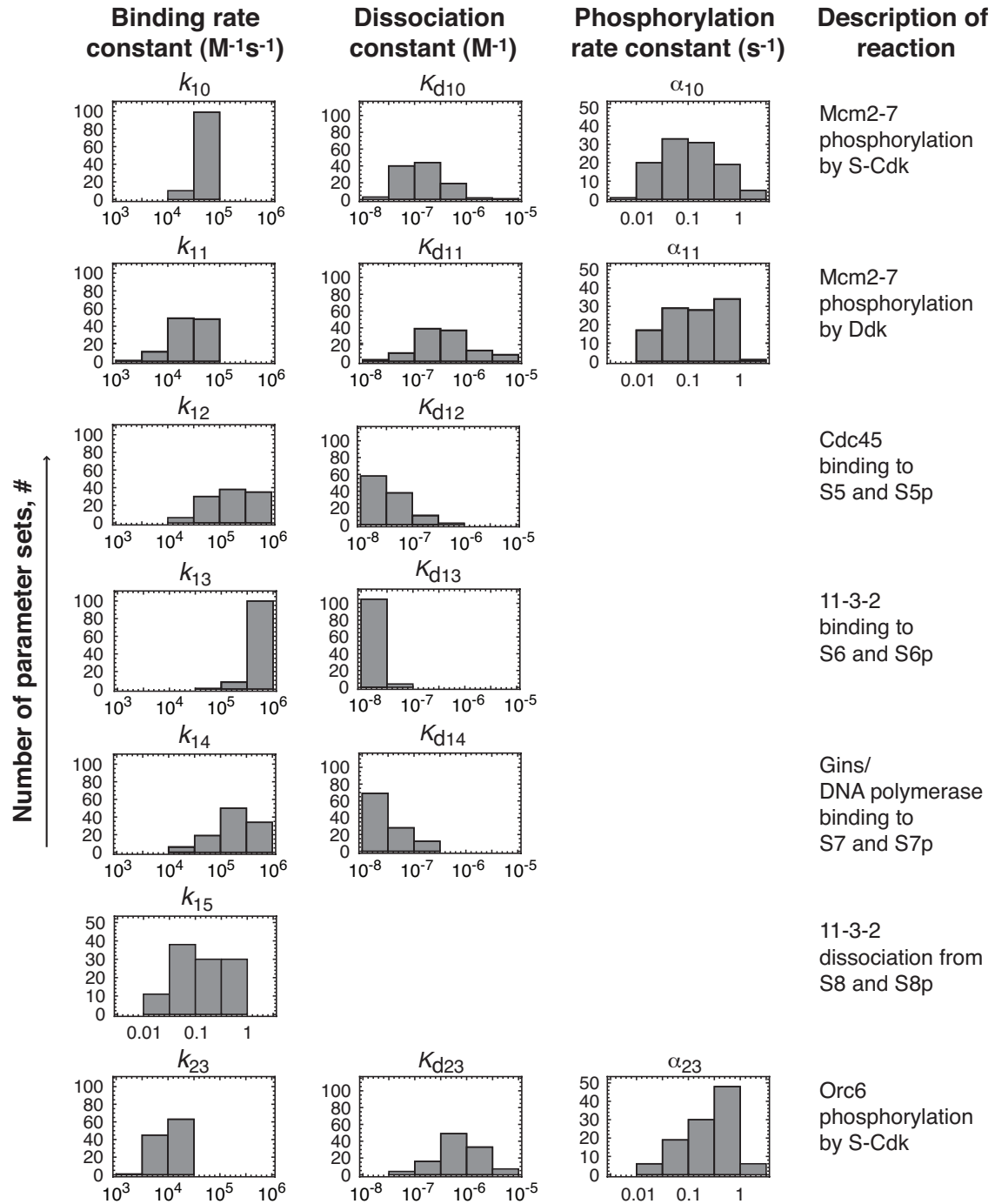

Supplement: Figure S1 — Distribution of optimized parameter values of the firing module (0.06 MB PDF) [file pcbi.1000783.s007.pdf]

Supporting Figure 2: Distribution of optimized parameter values of the 11-3-2 activator module

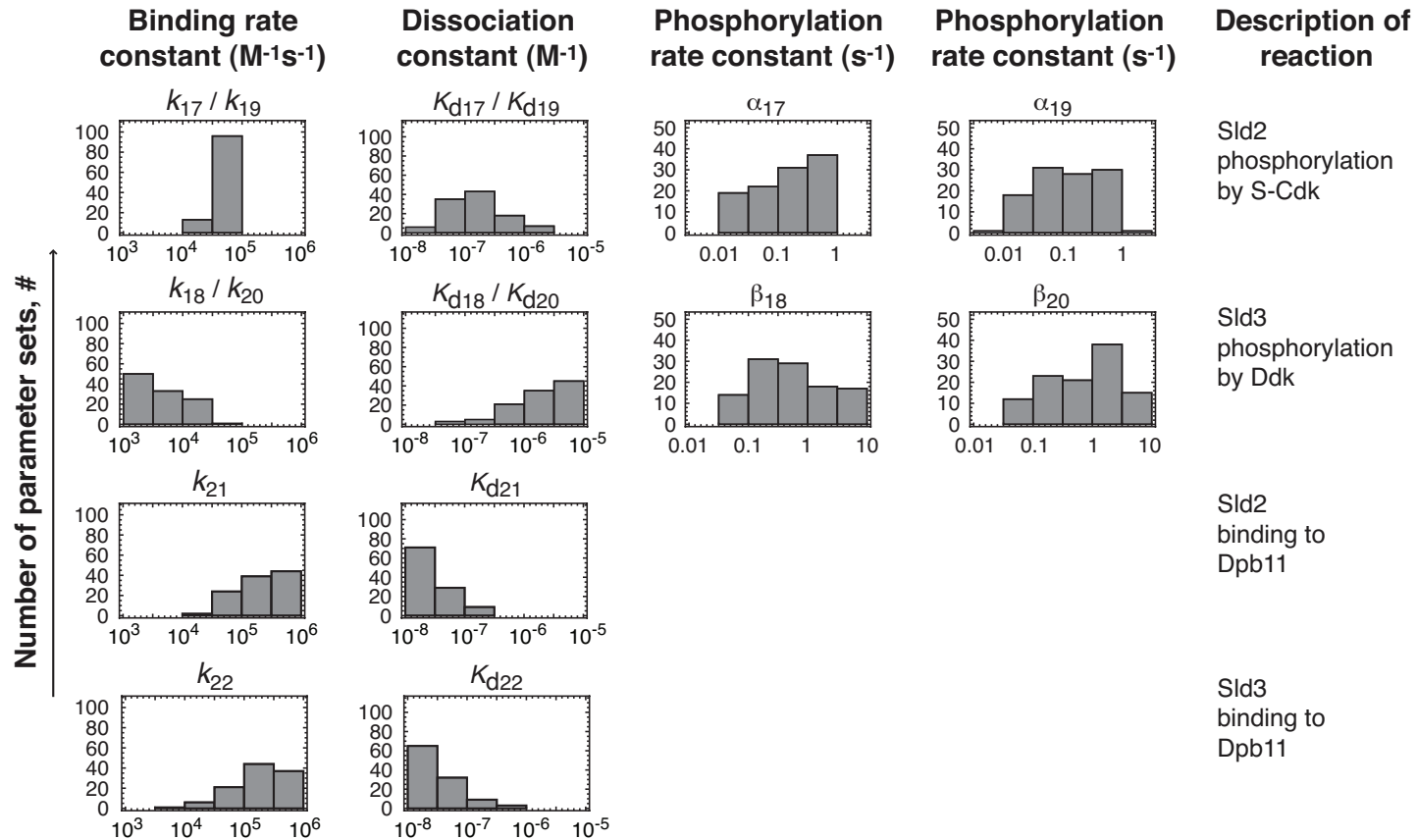

Supplement: Figure S2 — Distribution of optimized parameter values of the 11-3-2 activator module (0.05 MB PDF) [file pcbi.1000783.s008.pdf]
