# Supplementary material for: Mathematical Modelling of DNA Replication Reveals a Trade-off between Coherence of Origin Activation and Robustness against Rereplication
Source: PLoS Comput Biol. 2010 May 13;6(5):e1000783. doi: 10.1371/journal.pcbi.1000783 (PMC2869307; doi:10.1371/journal.pcbi.1000783)
Supplement: Figure S3 — Control coefficients of the kinetic parameters on the systems properties N, ρ, τ and Δ calculated with all generated parameter sets (0.05 MB PDF) [file pcbi.1000783.s009.pdf]

Supporting Figure 3: Control coefficients of the kinetic parameters on the systems properties N, ρ, τ and Δ calculated with all generated parameter sets

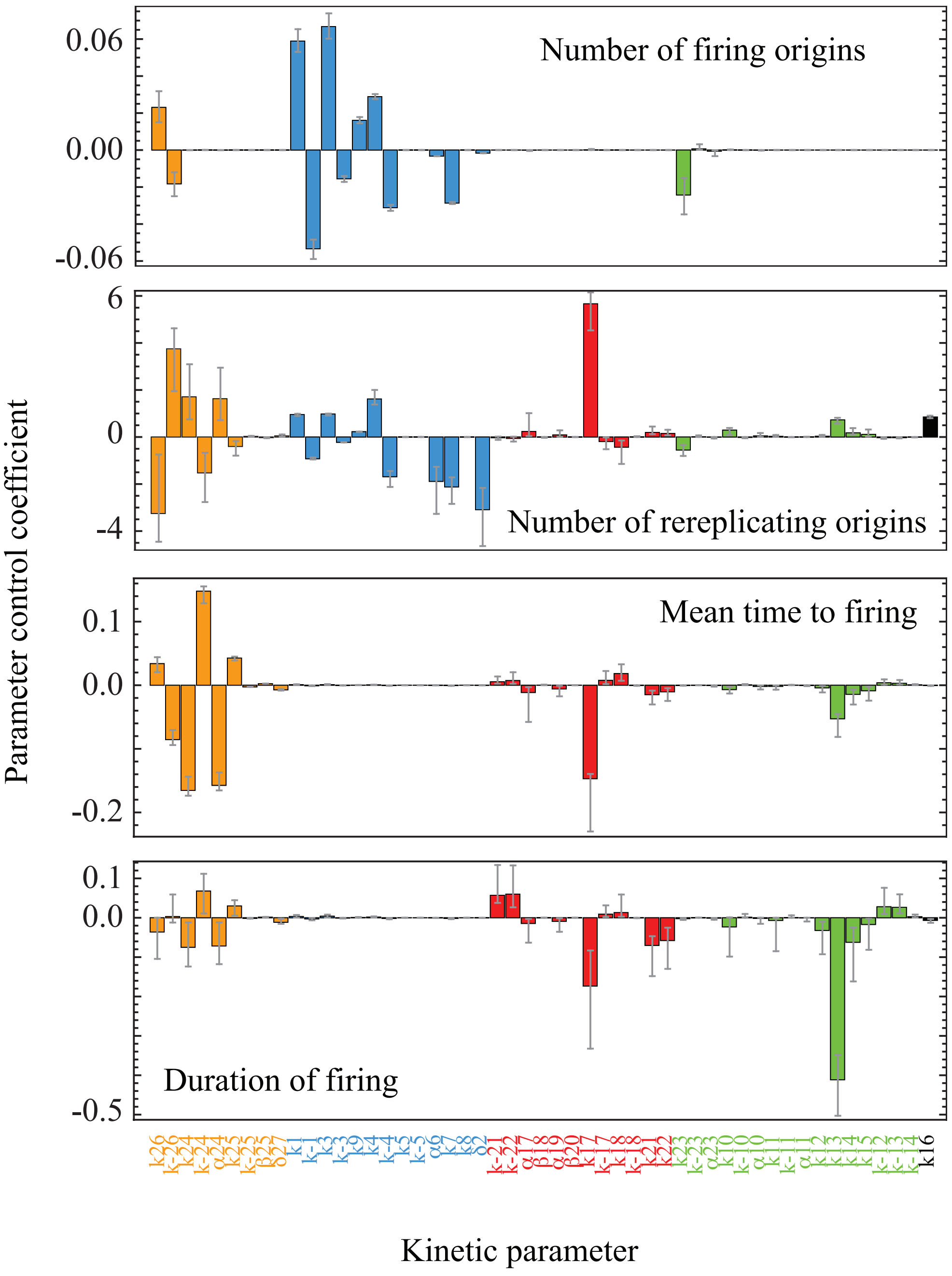

Shown is the mean value and standard deviation of control coefficients calculated for all optimized parameter sets.
