# Supplementary material for: Mathematical Modelling of DNA Replication Reveals a Trade-off between Coherence of Origin Activation and Robustness against Rereplication
Source: PLoS Comput Biol. 2010 May 13;6(5):e1000783. doi: 10.1371/journal.pcbi.1000783 (PMC2869307; doi:10.1371/journal.pcbi.1000783)
Supplement: Figure S4 — Initiation of DNA replication without catalytic replacement of the 11-3-2 activator complex (0.04 MB PDF) [file pcbi.1000783.s010.pdf]

Supporting Figure 4: Initiation of DNA replication without catalytic replacement of the 11-3-2 activator complex

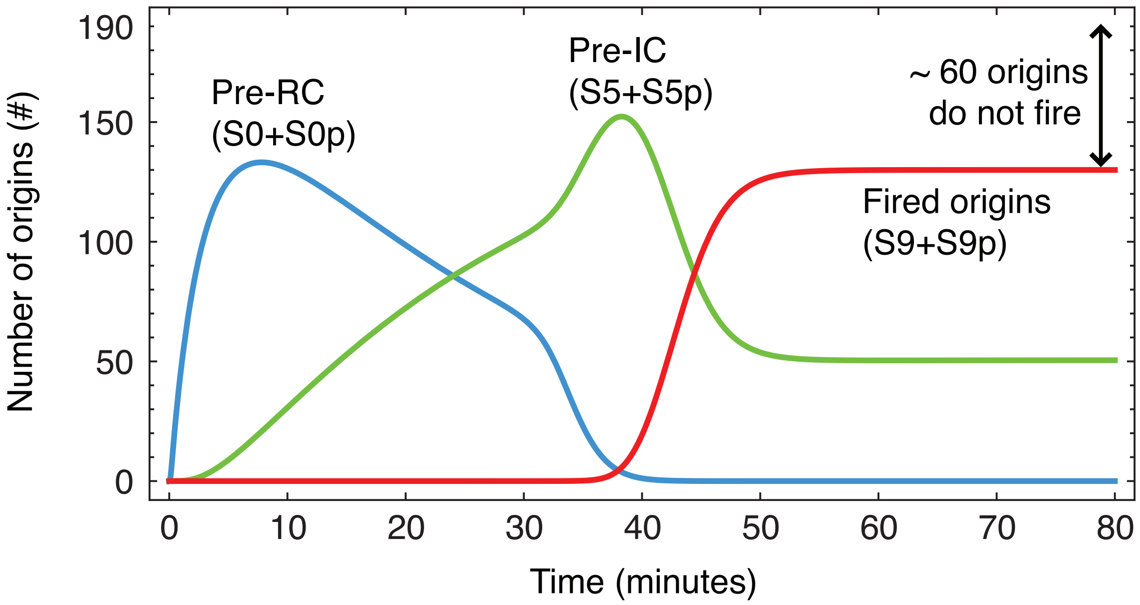

Incomplete initiation of replication origins would result, if the 11-3-2 activator complex remains associated with the replication origins, because the number of the 11-3-2 activator complexes in limited by the reported low number of Sld3, of only 130 molecules (Ghaemmaghami et al, 2003). A catalytic function of the 11-3-2 activator complex is proposed, guided by the experimental observation that the 11-3-2 activator complex is not part of the replisome and not needed for ongoing DNA synthesis. A replacement of the 11-3-2 activator complex from the origins after the loading of the GINS complex associated with the DNA polymerase allows a recycling and the use of one 11-3-2 activator complex at seversal origins.
