# Supplementary material for: Mathematical Modelling of DNA Replication Reveals a Trade-off between Coherence of Origin Activation and Robustness against Rereplication
Source: PLoS Comput Biol. 2010 May 13;6(5):e1000783. doi: 10.1371/journal.pcbi.1000783 (PMC2869307; doi:10.1371/journal.pcbi.1000783)
Supplement: Figure S5 — Dependence of the systems properties on the initial number of early origins calculated with the reference parameter set (0.03 MB PDF) [file pcbi.1000783.s011.pdf]

Supporting Figure 5: Dependence of the systems properties on the initial number of early origins calculated with the reference parameter set

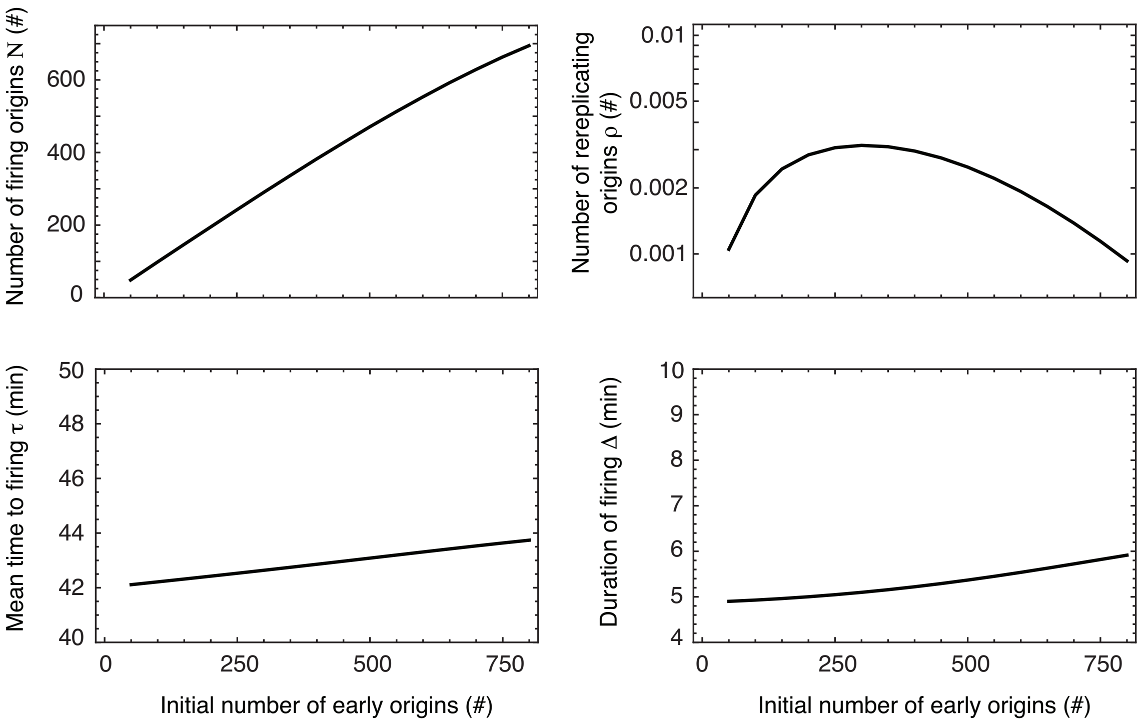

The initial number of early origins, used in the mathematical model, does not influence the values of the systems properties. The number of rereplicating origins, the mean time to firing and the duration of firing remain almost unchanges, when the initial number of early origins is varied between 50 to 800 origins. The number of firing origins increases proportional to the initial number of early origins, so that finally alway a fraction of $> 95\%$ is activated.
