# Supplementary material for: Mathematical Modelling of DNA Replication Reveals a Trade-off between Coherence of Origin Activation and Robustness against Rereplication
Source: PLoS Comput Biol. 2010 May 13;6(5):e1000783. doi: 10.1371/journal.pcbi.1000783 (PMC2869307; doi:10.1371/journal.pcbi.1000783)
Supplement: Figure S6 — Average fraction of activated origins and average number of rereplicating origins in parameter sets generated under different requirements for the constraints Nmin and ρmax. (0.04 MB PDF) [file pcbi.1000783.s012.pdf]

Supporting Figure 6: Average fraction of activated origins and average number of rereplicating origins in parameter sets generated under different requirements for the constraints N_{min} and ρ_{max}.

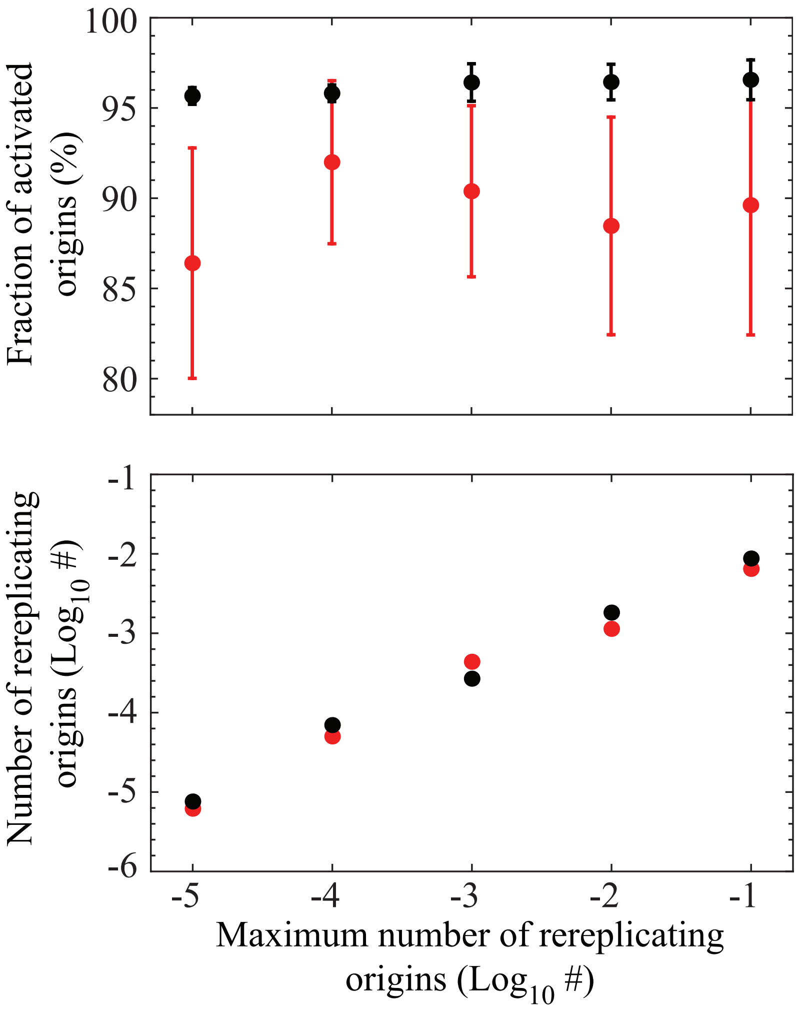

The maximum allowed number of rereplicating origins is varied between $\rho < 10^{-5}$ and $\rho < 10^{-1}$ and the minimal required fraction of activated origins distinguishes $N > 95\%$ (black dots) and $N > 50\%$ (red dots). Approximately 10 parameter sets are generated for each of combination of conditions. The relaxed requirements for the number of activated origins of 50%, as well as the relaxed requirement for the number of rereplicating origins of 10^{-1} is never exhausted. But the more stringent the requirement for both systems properties, the more these are exploited.
