# Supplementary material for: Mathematical Modelling of DNA Replication Reveals a Trade-off between Coherence of Origin Activation and Robustness against Rereplication
Source: PLoS Comput Biol. 2010 May 13;6(5):e1000783. doi: 10.1371/journal.pcbi.1000783 (PMC2869307; doi:10.1371/journal.pcbi.1000783)
Supplement: Figure S7 — Estimated number of replicated origins in dependence of the coherence of origin firing (0.03 MB PDF) [file pcbi.1000783.s013.pdf]

## Supporting Figure 7: Estimated number of passively replicated origins in dependence of the coherence of origin firing.

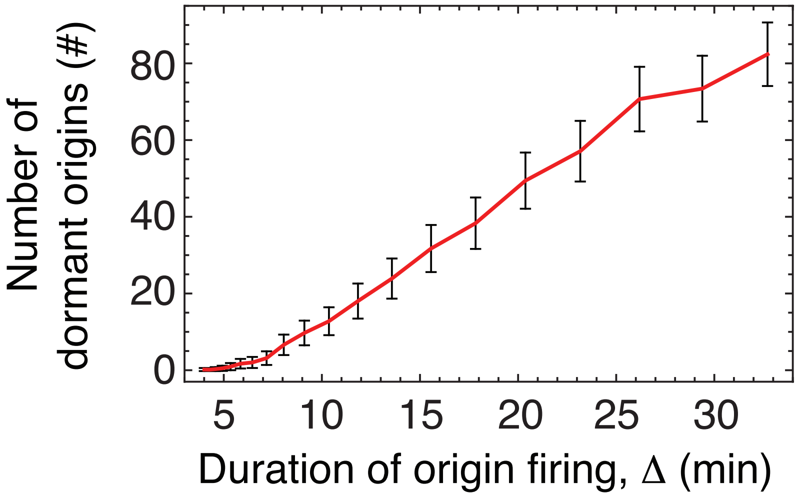


The number of passively replicated origins (also called dormant origins) increases with a prolonged duration of origin firing, that is a decreasing coherence between origin firing events. The number of dormant origins was calculated under the assumption that all 190 early origins considered here are positioned in the genome in one big connected cluster. The estimated number of dormant origins represents an average over 1000 calculations, each performed with varying distances between neighboring origins, which are randomly picked from the measured distribution of inter-origin-distances (Lengronne et al., 2001), as well as varying firing times, which are randomly picked from the firing rate derived from the mathematical model (Figure 1B of the main text).
