# Supplementary material for: Mathematical Modelling of DNA Replication Reveals a Trade-off between Coherence of Origin Activation and Robustness against Rereplication
Source: PLoS Comput Biol. 2010 May 13;6(5):e1000783. doi: 10.1371/journal.pcbi.1000783 (PMC2869307; doi:10.1371/journal.pcbi.1000783)
Supplement: Figure S8 — Firing rate for different S-Cdk concentrations (0.04 MB PDF) [file pcbi.1000783.s014.pdf]

Supporting Figure 8: Firing rate under different S-Cdk concentrations.

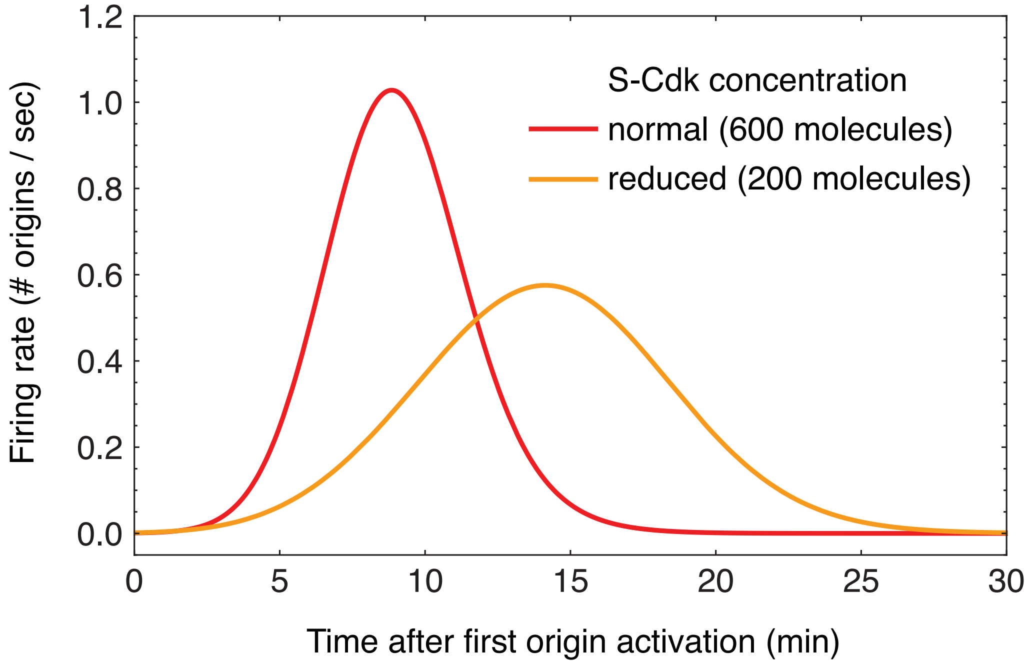

A short duration of origins firing is robustly obtained for a wide range of S-Cdk concentrations.
